# Supplementary material for: Nests of red wood ants (Formica rufa-group) are positively associated with tectonic faults: a double-blind test
Source: PeerJ. 2017 Oct 12;5:e3903. doi: 10.7717/peerj.3903 (PMC5641425; doi:10.7717/peerj.3903)
Supplement: Supplemental Information 1 — Knitted R markdown file showing all analyses. [file peerj-05-3903-s001.pdf]

Ants and Faults Analyses

AME & IDT & RRR

August 30, 2017

This is the reproducible code for analyses and figures: Make sure you have a reliable internet connection to download some of the spatial data needed for mapping.

Load required libraries and setwd

```
library(raster)

## Loading required package: sp

## Warning: package 'sp' was built under R version 3.4.1

library(rgdal)

## Warning: package 'rgdal' was built under R version 3.4.1

## rgdal: version: 1.2-8, (SVN revision 663)
##  Geospatial Data Abstraction Library extensions to R successfully loaded
##  Loaded GDAL runtime: GDAL 2.1.3, released 2017/20/01
##  Path to GDAL shared files:
/Library/Frameworks/R.framework/Versions/3.4/Resources/library/rgdal/gdal
##  Loaded PROJ.4 runtime: Rel. 4.9.3, 15 August 2016, [PJ_VERSION: 493]
##  Path to PROJ.4 shared files:
/Library/Frameworks/R.framework/Versions/3.4/Resources/library/rgdal/proj
##  Linking to sp version: 1.2-4

library(ggplot2)
library (ggmap)
library(reshape2)
library(plyr)
library (gridExtra)
library(spatstat)

## Warning: package 'spatstat' was built under R version 3.4.1

## Loading required package: nlme

##
## Attaching package: 'nlme'

## The following object is masked from 'package:raster':
##
##     getData

## Loading required package: rpart
```

```
##
## spatstat 1.52-1       (nickname: 'Apophenia')
## For an introduction to spatstat, type 'beginner'

##
## Attaching package: 'spatstat'

## The following objects are masked from 'package:raster':
##
##     area, rotate, shift

setwd("/Users/israel/Desktop/OneDrive - Lawrence University/DK Ants")
```

Read in data

```
dk.ants <- read.csv("DK_RWAnts.csv") #this reads in the data and assigns it
to "dk.ants"
dk.faults <- read.csv("Faults_10_26.csv", header=T) #this is the file for
geological faults
summary(dk.faults)

##        Id       x_start          y_start          x_end
##  Min.   :0   Min.   : 7.711   Min.   :55.43   Min.   : 7.635
##  1st Qu.:0   1st Qu.: 8.552   1st Qu.:56.41   1st Qu.: 8.583
##  Median :0   Median : 8.969   Median :56.77   Median : 9.013
##  Mean   :0   Mean   : 9.071   Mean   :56.74   Mean   : 9.106
##  3rd Qu.:0   3rd Qu.: 9.483   3rd Qu.:57.07   3rd Qu.: 9.529
##  Max.   :0   Max.   :10.973   Max.   :57.90   Max.   :10.950
##
##      y_end              Fault_Type             Fault_Age     Fault_Tren
##  Min.   :55.44   Fault        :215   Pre-Quaternary: 38   NW-SE   :228
##  1st Qu.:56.40   Major Fault :238   NA's          :564   WNW-ESE:140
##  Median :56.74   Normal Fault:149                          NNW-SSE: 93
##  Mean   :56.73                                             NE-SW  : 88
##  3rd Qu.:57.05                                             NNE-SSW: 40
##  Max.   :57.90                                             ENE-WSW:  7
##                                                            (Other):  6
##    Direction                                       Author
##  Min.   :-348.8   2015 GEUS Server                  :179
##  1st Qu.:-326.2   2010 Pedersen-Gravensen           : 54
##  Median :-326.2   2010 Pedersen Gravesen            : 38
##  Mean   :-261.6   1997 Veijbaek/2008 Petersen et al.:  1
##  3rd Qu.:-303.8   1998 Veijbaek/2008 Petersen et al.:  1
##  Max.   :   0.0   1999 Veijbaek/2008 Petersen et al.:  1
##                   (Other)                           :328
```

So now we have two data files, dk.ants with locations and species of ants, and dk.faults with start and endpoints of each fault

Let's start with some simple plotting: We can plot the detailed distribution of RWA nests and absence points for each of the two study regions

```
## Map from URL : http://tile.stamen.com/toner/10/535/312.png
## Map from URL : http://tile.stamen.com/toner/10/536/312.png
## Map from URL : http://tile.stamen.com/toner/10/537/312.png
## Map from URL : http://tile.stamen.com/toner/10/535/313.png
## Map from URL : http://tile.stamen.com/toner/10/536/313.png
## Map from URL : http://tile.stamen.com/toner/10/537/313.png
## Map from URL : http://tile.stamen.com/toner/10/535/314.png
## Map from URL : http://tile.stamen.com/toner/10/536/314.png
## Map from URL : http://tile.stamen.com/toner/10/537/314.png
```

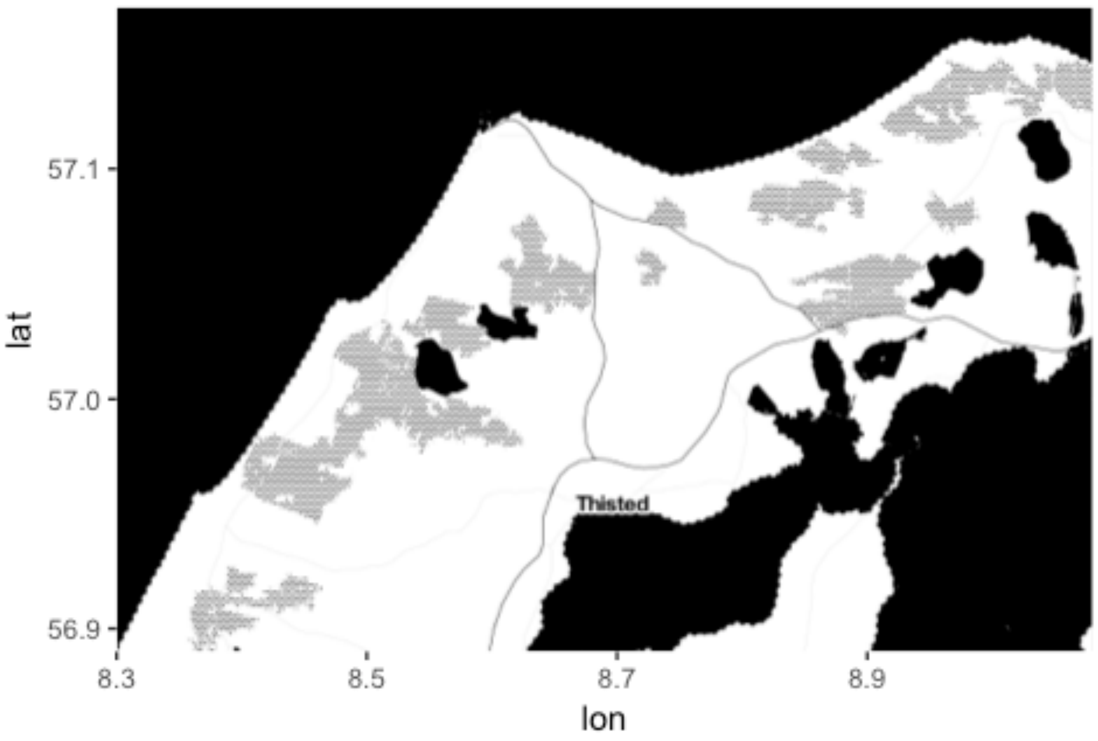


```
## Map from URL : http://tile.stamen.com/toner/10/535/315.png
## Map from URL : http://tile.stamen.com/toner/10/536/315.png
## Map from URL : http://tile.stamen.com/toner/10/535/316.png
## Map from URL : http://tile.stamen.com/toner/10/536/316.png
```

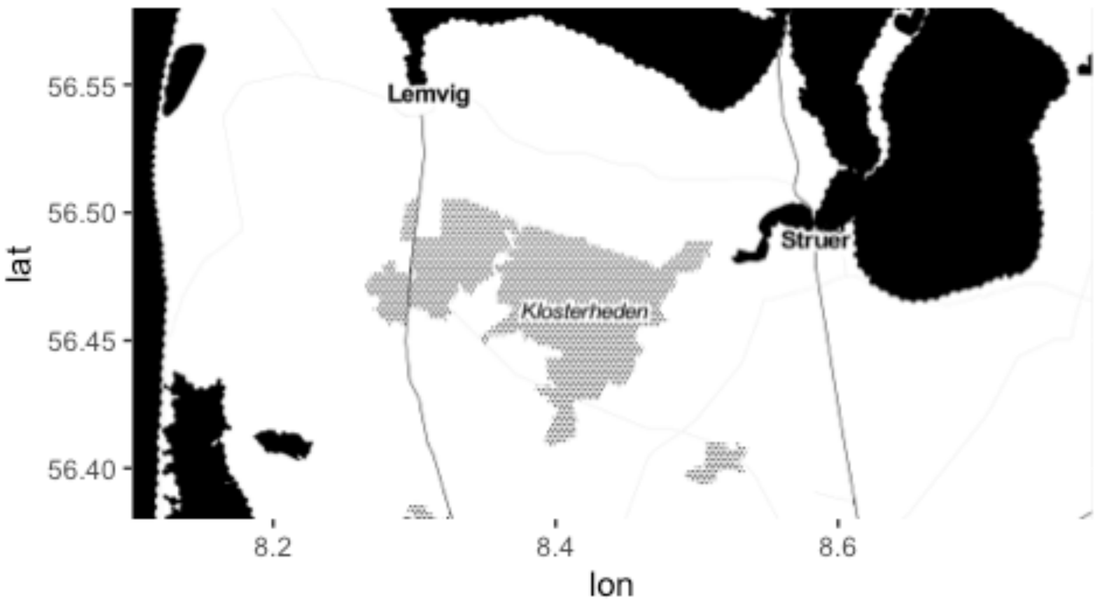


```
## Warning: Removed 87 rows containing missing values (geom_point).

## Warning: Removed 211 rows containing missing values (geom_point).
```

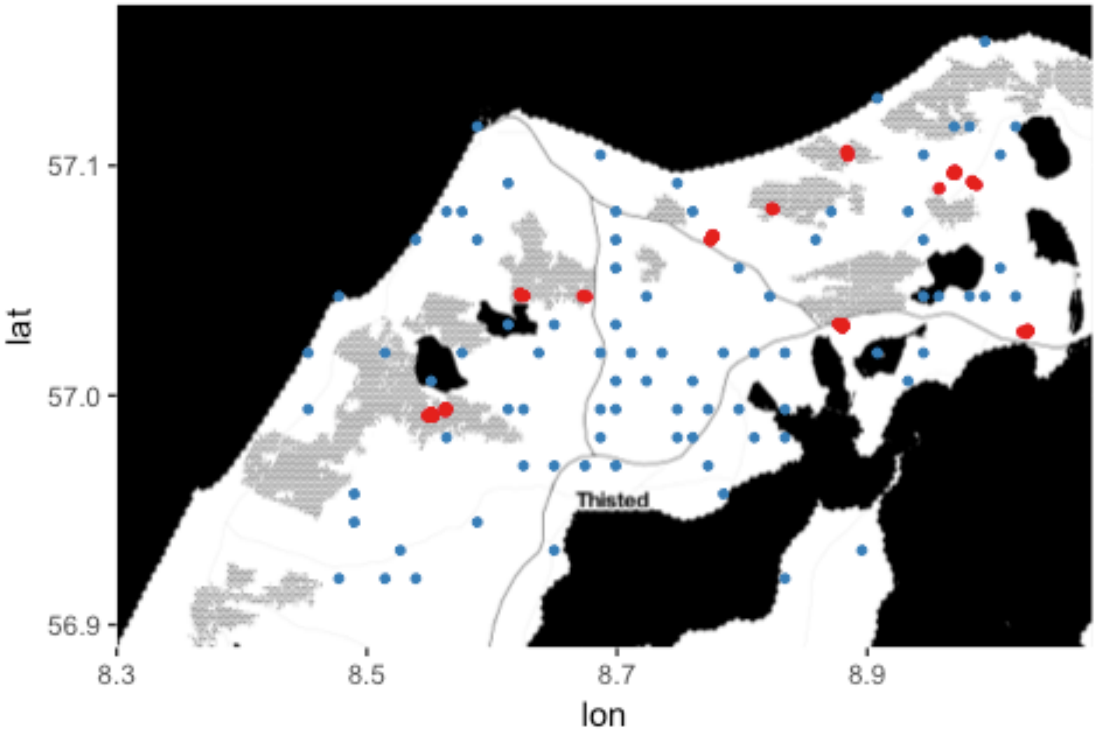


```
## Warning: Removed 84 rows containing missing values (geom_point).

## Warning: Removed 71 rows containing missing values (geom_point).
```

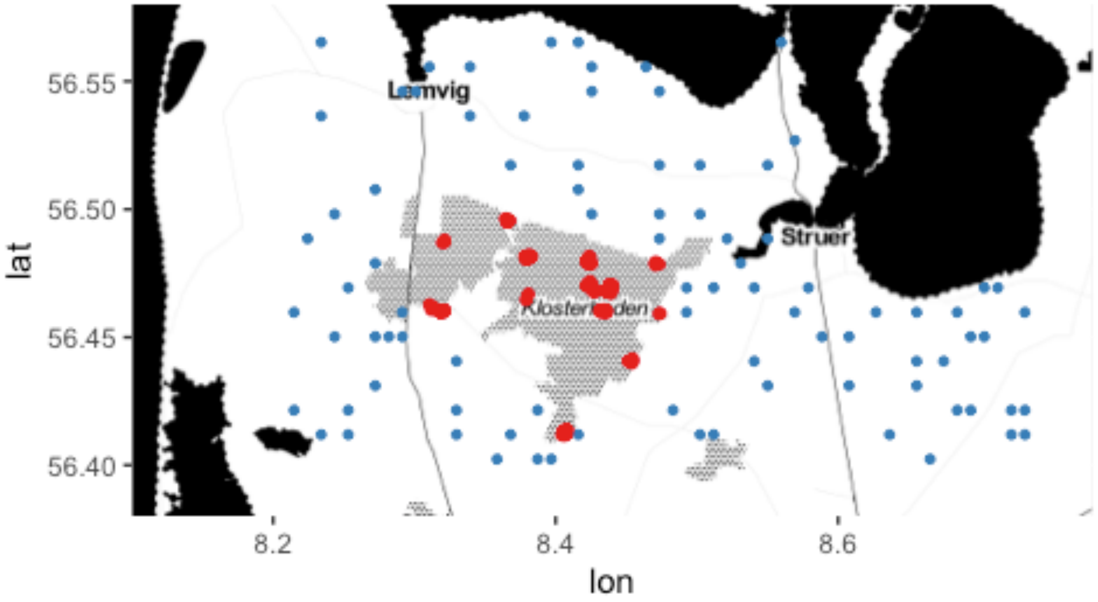

We can also map the distribution of the faults in the entire Jutland Penninsula

```
#study region boud.box= xmin=8.115, xmax=9.129, ymin=56.3, ymax=57.25
region_base = get_map(location=c( 8.05, 55, 11,  58), maptype="toner-
background")

## maptype = "toner-background" is only available with source = "stamen".

## resetting to source = "stamen"...

## Map from URL : http://tile.stamen.com/toner-background/8/133/77.png

## Map from URL : http://tile.stamen.com/toner-background/8/134/77.png

## Map from URL : http://tile.stamen.com/toner-background/8/135/77.png

## Map from URL : http://tile.stamen.com/toner-background/8/133/78.png

## Map from URL : http://tile.stamen.com/toner-background/8/134/78.png

## Map from URL : http://tile.stamen.com/toner-background/8/135/78.png

## Map from URL : http://tile.stamen.com/toner-background/8/133/79.png

## Map from URL : http://tile.stamen.com/toner-background/8/134/79.png
```

```
## Map from URL : http://tile.stamen.com/toner-background/8/135/79.png

## Map from URL : http://tile.stamen.com/toner-background/8/133/80.png

## Map from URL : http://tile.stamen.com/toner-background/8/134/80.png

## Map from URL : http://tile.stamen.com/toner-background/8/135/80.png

map3<-ggmap(region_base)
map3<- map3 +  geom_point(data=abs_pts, aes(x=x, y=y), cex=1, col="#377eb8",
alpha=0.5 ) +
  geom_point(data=ant_pts, aes(x=x, y=y), cex=1, col="#e41a1c", alpha=0.5 )
+
  geom_segment(aes(x = x_start, y = y_start, xend = x_end, yend = y_end),
data = dk.faults,
               color= "#756bb1", cex=.75)
map3

## Warning: Removed 37 rows containing missing values (geom_segment).
```

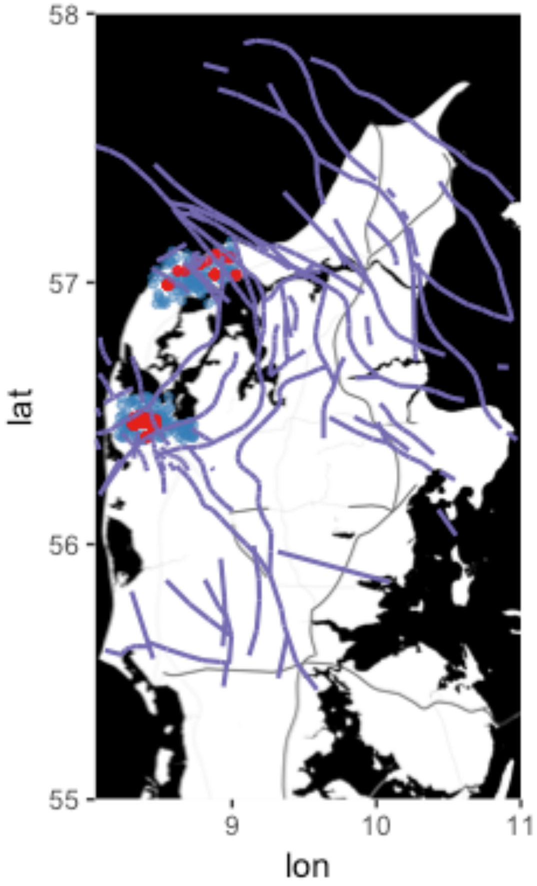

Now we can test for spatial clustering of the RWA nest points in both of the study region: The extent of the fault dataset is much larger than the extent of the ant data. And the ant data come in two widely separated clusters. So let's make some subsets to work with.

We could read off the graph, or subset by hand, or... but all of those are tedious. Let's use some of the facilities within spatstat instead.

```
#Start by identifying the extent ("window") of the ant data
dk.owin.ants <- owin(c(min(dk.ants$x)-.05, max(dk.ants$x)+.05),
c(min(dk.ants$y)-.05, max(dk.ants$y)+.05) )

#create a "point pattern object" out of dk.ants
dk.ppp.ants <- ppp(dk.ants$x, dk.ants$y, window=dk.owin.ants,
marks=dk.ants$SP_ID)

#now creat a "point segment object" out of dk.faults
dk.owin.faults <- owin(c(min(dk.faults$x_start, dk.faults$x_end),
max(dk.faults$x_start, dk.faults$x_end)), c(min(dk.faults$y_start,
dk.faults$y_end),    max(dk.faults$y_start, dk.faults$y_end)))

dk.psp.faults <- psp(dk.faults$x_start, dk.faults$y_start, dk.faults$x_end,
dk.faults$y_end, window=dk.owin.faults)

dk.psp.faults.1 <- dk.psp.faults[dk.owin.ants]
```

So now we have two spatial data files that we can work with: dk.ppp.ants and dk.psp.faults.1

```
str(dk.ppp.ants)

## List of 6
##  $ window    :List of 4
##   ..$ type  : chr "rectangle"
##   ..$ xrange: num [1:2] 8.16 9.08
##   ..$ yrange: num [1:2] 56.4 57.2
##   ..$ units :List of 3
##   .. ..$ singular  : chr "unit"
##   .. ..$ plural    : chr "units"
##   .. ..$ multiplier: num 1
##   .. ..- attr(*, "class")= chr "units"
##   ..- attr(*, "class")= chr "owin"
##  $ n         : int 453
##  $ x         : num [1:453] 8.21 8.21 8.22 8.23 8.23 ...
##  $ y         : num [1:453] 56.5 56.4 56.5 56.6 56.5 ...
##  $ markformat: chr "vector"
##  $ marks     : Factor w/ 6 levels "","fusca","none",..: 3 3 3 3 3 3 3 3 3
3 ...
##  - attr(*, "class")= chr "ppp"

str(dk.psp.faults.1)

## List of 4
##  $ ends      :'data.frame':  187 obs. of  4 variables:
##   ..$ x0: num [1:187] 8.37 8.54 8.81 8.48 8.72 ...
##   ..$ y0: num [1:187] 56.6 56.4 56.4 57.1 57.1 ...
```

```
##   ..$ x1: num [1:187] 8.38 8.56 8.85 8.53 8.71 ...
##   ..$ y1: num [1:187] 56.5 56.4 56.4 57.1 57.1 ...
##  $ window    :List of 4
##   ..$ type  : chr "rectangle"
##   ..$ xrange: num [1:2] 8.16 9.08
##   ..$ yrange: num [1:2] 56.4 57.2
##   ..$ units :List of 3
##   .. ..$ singular  : chr "unit"
##   .. ..$ plural    : chr "units"
##   .. ..$ multiplier: num 1
##   .. ..- attr(*, "class")= chr "units"
##   ..- attr(*, "class")= chr "owin"
##  $ n         : int 187
##  $ markformat: chr "none"
##  - attr(*, "class")= chr [1:2] "psp" "list"

#Are ants randomly distributed?

Kest(dk.ppp.ants[dk.ppp.ants$marks != "none"])

## Function value object (class 'fv')
## for the function r -> K(r)
## .................................................................
##        Math.label       Description
## r      r                distance argument r
## theo   K[pois](r)       theoretical Poisson K(r)
## border hat(K)[bord](r)  border-corrected estimate of K(r)
## trans  hat(K)[trans](r) translation-corrected estimate of K(r)
## iso    hat(K)[iso](r)   isotropic-corrected estimate of K(r)
## .................................................................
## Default plot formula:  .~r
## where "." stands for 'iso', 'trans', 'border', 'theo'
## Recommended range of argument r: [0, 0.21288]
## Available range of argument r: [0, 0.21288]

plot(Kest(dk.ppp.ants[dk.ppp.ants$marks != "none"])) #also illustrates nested
functions
```

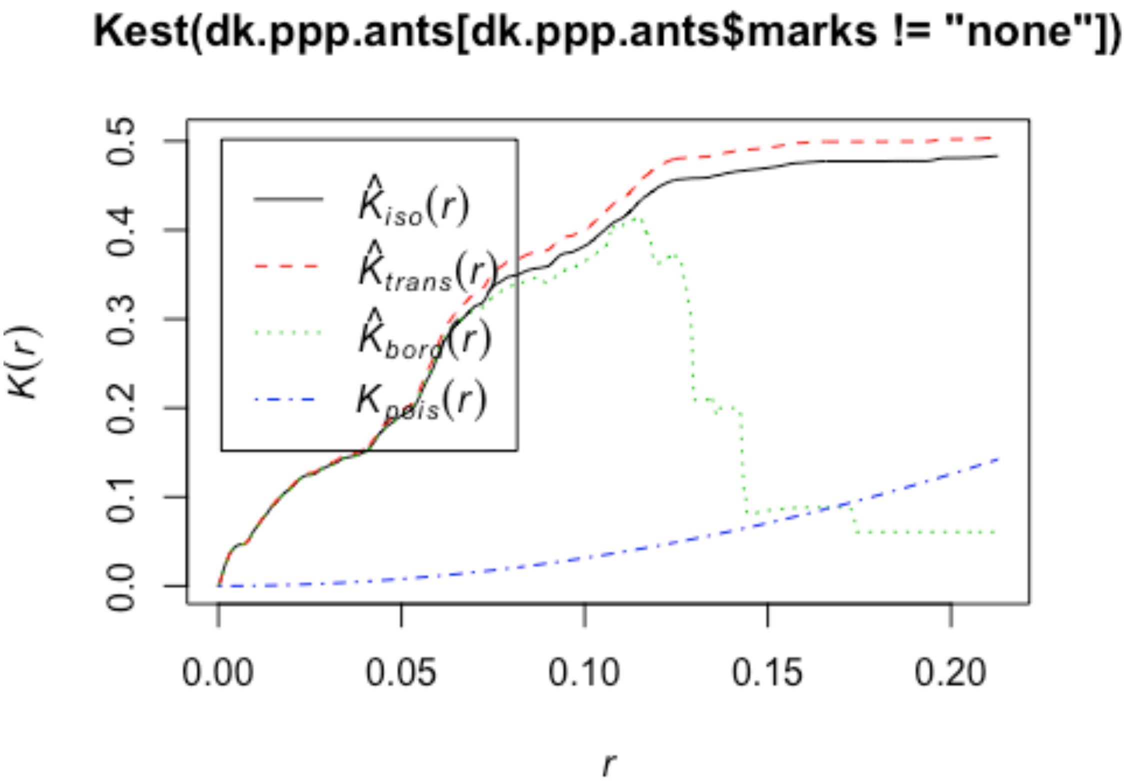


```
plot(envelope(dk.ppp.ants[dk.ppp.ants$marks != "none"], fun=Kest, nsim=99))

## Generating 99 simulations of CSR  ...
## 1, 2, 3, 4, 5, 6, 7, 8, 9, 10, 11, 12, 13, 14, 15, 16, 17, 18, 19, 20, 21,
22, 23, 24, 25, 26, 27, 28, 29, 30, 31, 32, 33, 34, 35, 36, 37, 38,
## 39, 40, 41, 42, 43, 44, 45, 46, 47, 48, 49, 50, 51, 52, 53, 54, 55, 56,
57, 58, 59, 60, 61, 62, 63, 64, 65, 66, 67, 68, 69, 70, 71, 72, 73, 74, 75,
76,
## 77, 78, 79, 80, 81, 82, 83, 84, 85, 86, 87, 88, 89, 90, 91, 92, 93, 94,
95, 96, 97, 98,  99.
##
## Done.
```

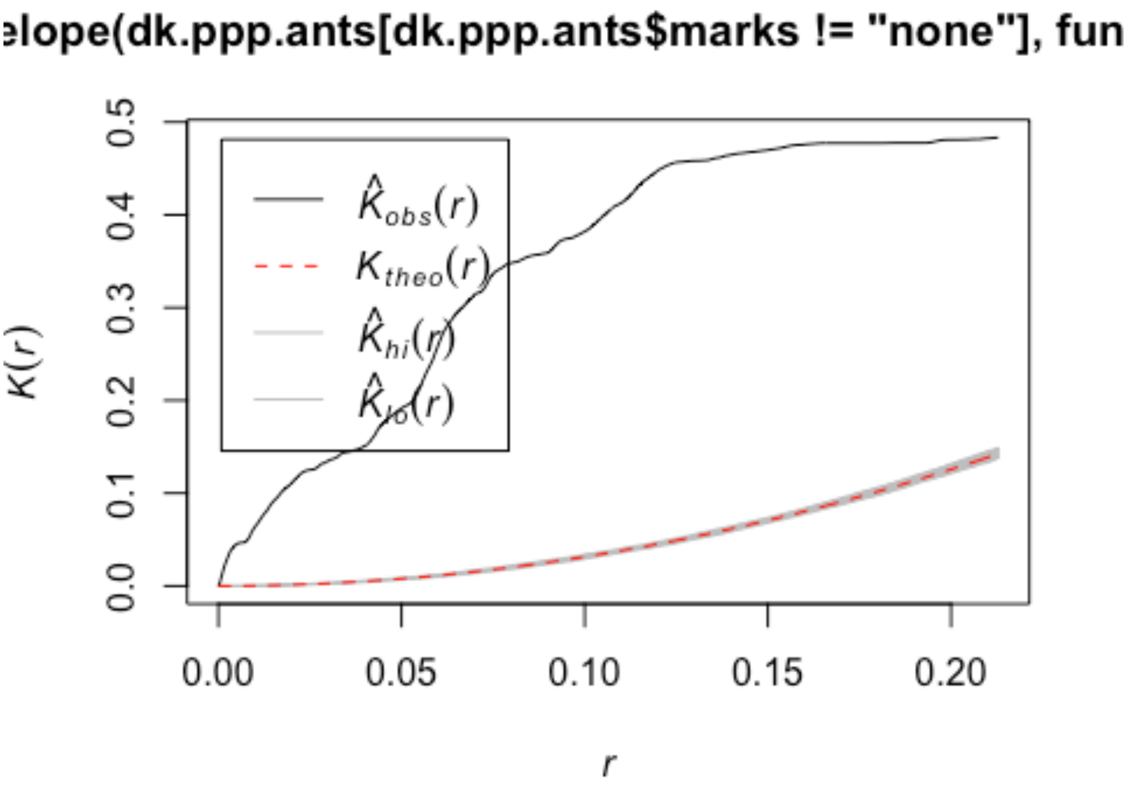

Aside: Ants are clumped, but is it because we have two clumps? Maybe we should just look at one clump?

```
#reminder What would a random pattern look like?

nX <-npoints(dk.ppp.ants[dk.ppp.ants$marks != "none"]) #same number of points
as ants
nX

## [1] 282

PatList <- runifpoint(nX) #generates using runifpoint function, maintains
owin
plot(PatList)
```

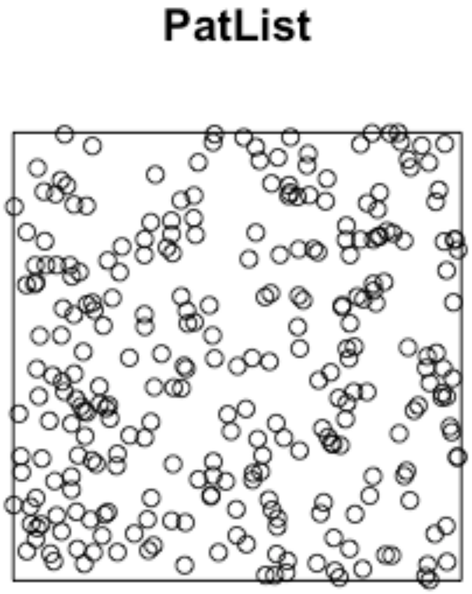


```
plot(envelope(PatList, fun=Kest, nsim=99))
```

```
## Generating 99 simulations of CSR  ...
## 1, 2, 3, 4, 5, 6, 7, 8, 9, 10, 11, 12, 13, 14, 15, 16, 17, 18, 19, 20, 21,
22, 23, 24, 25, 26, 27, 28, 29, 30, 31, 32, 33, 34, 35, 36, 37, 38,
## 39, 40, 41, 42, 43, 44, 45, 46, 47, 48, 49, 50, 51, 52, 53, 54, 55, 56,
57, 58, 59, 60, 61, 62, 63, 64, 65, 66, 67, 68, 69, 70, 71, 72, 73, 74, 75,
76,
## 77, 78, 79, 80, 81, 82, 83, 84, 85, 86, 87, 88, 89, 90, 91, 92, 93, 94,
95, 96, 97, 98,  99.
##
## Done.
```

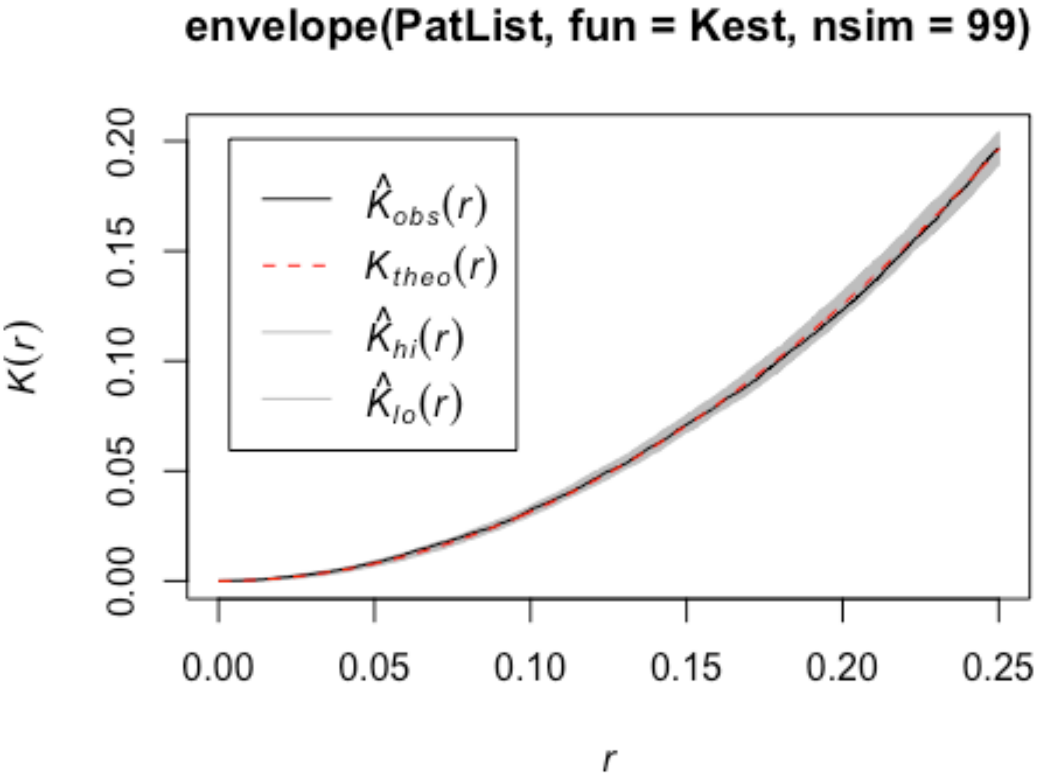


```
par(mfrow=c(1,2))
plot(envelope(dk.ppp.ants[dk.ppp.ants$marks != "none"], fun=Kest, nsim=99),
main="ants")
```

```
## Generating 99 simulations of CSR  ...
## 1, 2, 3, 4, 5, 6, 7, 8, 9, 10, 11, 12, 13, 14, 15, 16, 17, 18, 19, 20, 21,
22, 23, 24, 25, 26, 27, 28, 29, 30, 31, 32, 33, 34, 35, 36, 37, 38,
## 39, 40, 41, 42, 43, 44, 45, 46, 47, 48, 49, 50, 51, 52, 53, 54, 55, 56,
57, 58, 59, 60, 61, 62, 63, 64, 65, 66, 67, 68, 69, 70, 71, 72, 73, 74, 75,
76,
## 77, 78, 79, 80, 81, 82, 83, 84, 85, 86, 87, 88, 89, 90, 91, 92, 93, 94,
95, 96, 97, 98,  99.
##
## Done.
```

```
plot(envelope(rpoispp(273), fun=Kest, nsim=99), main="Poisson")
```

```
## Generating 99 simulations of CSR  ...
## 1, 2, 3, 4, 5, 6, 7, 8, 9, 10, 11, 12, 13, 14, 15, 16, 17, 18, 19, 20, 21,
22, 23, 24, 25, 26, 27, 28, 29, 30, 31, 32, 33, 34, 35, 36, 37, 38,
## 39, 40, 41, 42, 43, 44, 45, 46, 47, 48, 49, 50, 51, 52, 53, 54, 55, 56,
57, 58, 59, 60, 61, 62, 63, 64, 65, 66, 67, 68, 69, 70, 71, 72, 73, 74, 75,
76,
## 77, 78, 79, 80, 81, 82, 83, 84, 85, 86, 87, 88, 89, 90, 91, 92, 93, 94,
```

```
95, 96, 97, 98,  99.
##
## Done.
```

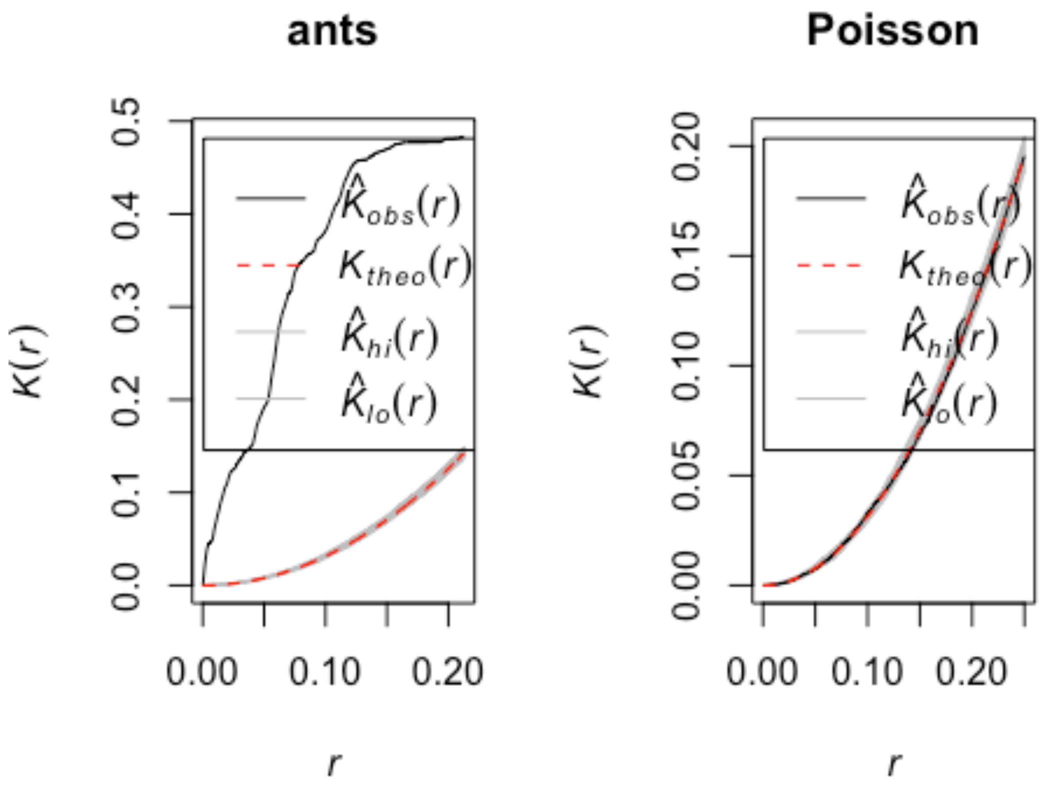


```
#smaller ant subset

#one way to identify groups
plot(dk.ants$x, dk.ants$y)
hist(dk.ants$y)
```

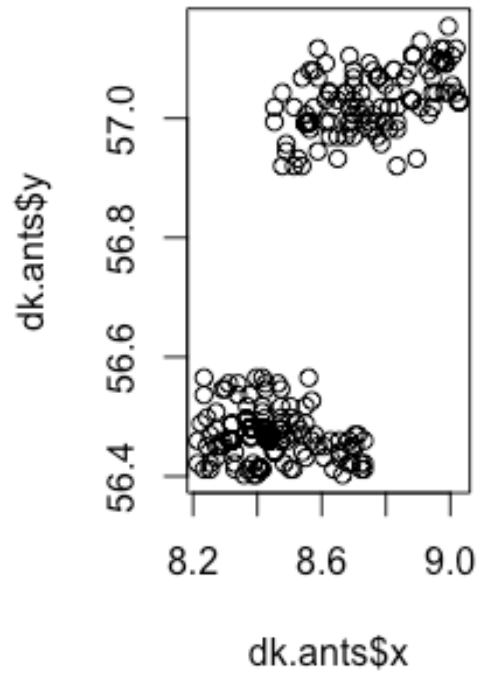

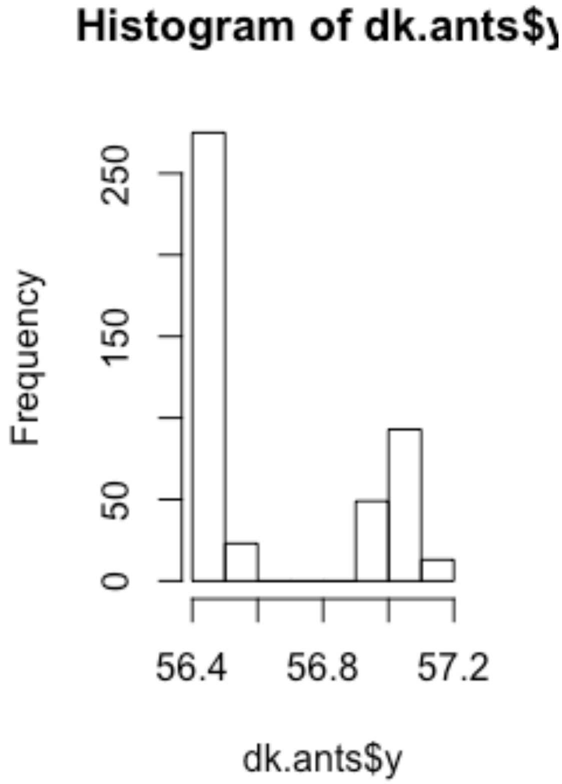


```
#clip it
str(dk.owin.ants)

## List of 4
##  $ type  : chr "rectangle"
##  $ xrange: num [1:2] 8.16 9.08
##  $ yrange: num [1:2] 56.4 57.2
##  $ units :List of 3
##   ..$ singular  : chr "unit"
##   ..$ plural    : chr "units"
##   ..$ multiplier: num 1
##   ..- attr(*, "class")= chr "units"
##  - attr(*, "class")= chr "owin"

dk.owin.ants.2 <- dk.owin.ants

#Thy clump
dk.owin.ants.2$yrange <- c(56.8, 57.2)
dk.owin.ants.2$xrange <-c(8.4, 9.08)

#Klosterhede clump
dk.owin.ants.2$yrange <- c(56.34, 56.6)
dk.owin.ants.2$xrange <-c(8.16, 8.8)
```

```
dk.ppp.ants.2 <- dk.ppp.ants[dk.owin.ants.2]
dk.psp.faults.2 <- dk.psp.faults.1[dk.owin.ants.2]

#plot it
par(mfrow=c(1,1))
plot(dk.psp.faults.2)
plot(dk.ppp.ants.2[dk.ppp.ants.2$marks =="none"], col="black", pch=1,
add=TRUE)
plot(unmark(dk.ppp.ants.2[dk.ppp.ants.2$marks !="none"]), col="red", pch=1,
add=TRUE)
```

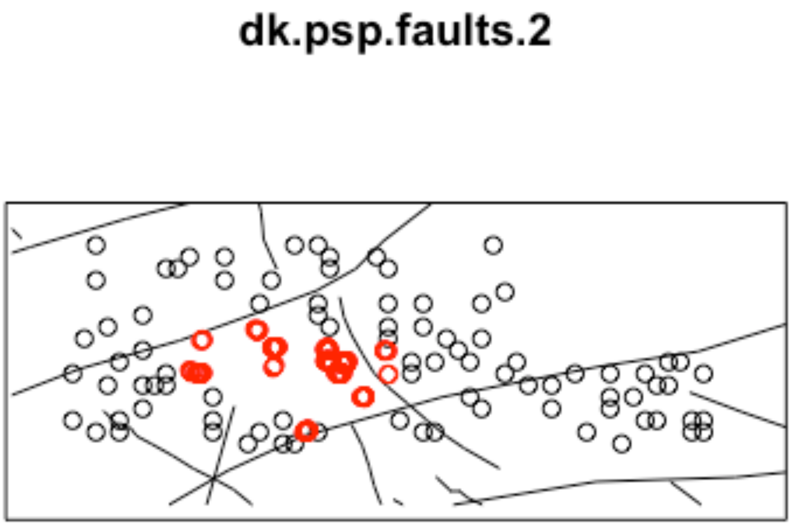


```
#test it
par(mfrow=c(1,2))
plot(envelope(dk.ppp.ants.2[dk.ppp.ants.2$marks !="none"], fun=Kest,
nsim=99), main="ants")

## Generating 99 simulations of CSR  ...
## 1, 2, 3, 4, 5, 6, 7, 8, 9, 10, 11, 12, 13, 14, 15, 16, 17, 18, 19, 20, 21,
22, 23, 24, 25, 26, 27, 28, 29, 30, 31, 32, 33, 34, 35, 36, 37, 38,
## 39, 40, 41, 42, 43, 44, 45, 46, 47, 48, 49, 50, 51, 52, 53, 54, 55, 56,
57, 58, 59, 60, 61, 62, 63, 64, 65, 66, 67, 68, 69, 70, 71, 72, 73, 74, 75,
76,
```

```
## 77, 78, 79, 80, 81, 82, 83, 84, 85, 86, 87, 88, 89, 90, 91, 92, 93, 94,
95, 96, 97, 98,  99.
##
## Done.
```

```
plot(envelope(dk.ppp.ants.2[dk.ppp.ants.2$marks =="none"], fun=Kest,
nsim=99), main="no ants")
```

```
## Generating 99 simulations of CSR  ...
## 1, 2, 3, 4, 5, 6, 7, 8, 9, 10, 11, 12, 13, 14, 15, 16, 17, 18, 19, 20, 21,
22, 23, 24, 25, 26, 27, 28, 29, 30, 31, 32, 33, 34, 35, 36, 37, 38,
## 39, 40, 41, 42, 43, 44, 45, 46, 47, 48, 49, 50, 51, 52, 53, 54, 55, 56,
57, 58, 59, 60, 61, 62, 63, 64, 65, 66, 67, 68, 69, 70, 71, 72, 73, 74, 75,
76,
## 77, 78, 79, 80, 81, 82, 83, 84, 85, 86, 87, 88, 89, 90, 91, 92, 93, 94,
95, 96, 97, 98,  99.
##
## Done.
```

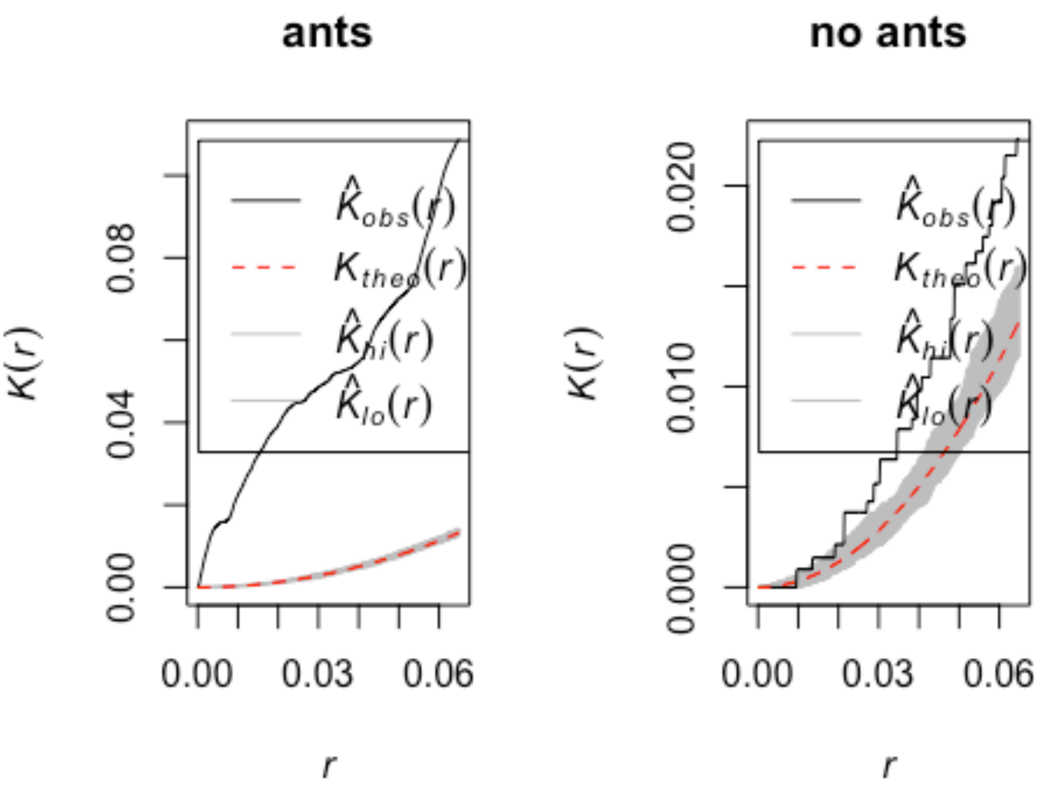

last but not least, let's look at relationships between ants and faults

```
dk.ppp.ants.2a <-unmark(dk.ppp.ants.2[dk.ppp.ants.2$marks != "none"])

dk.ppp.ants.2na <-unmark(dk.ppp.ants.2[dk.ppp.ants.2$marks == "none"])
```

```
par(mfrow=c(1,1))
plot(dk.ppp.ants.2a, pch=16, main="Danish ants")
plot(dk.psp.faults.2, add=TRUE)
```

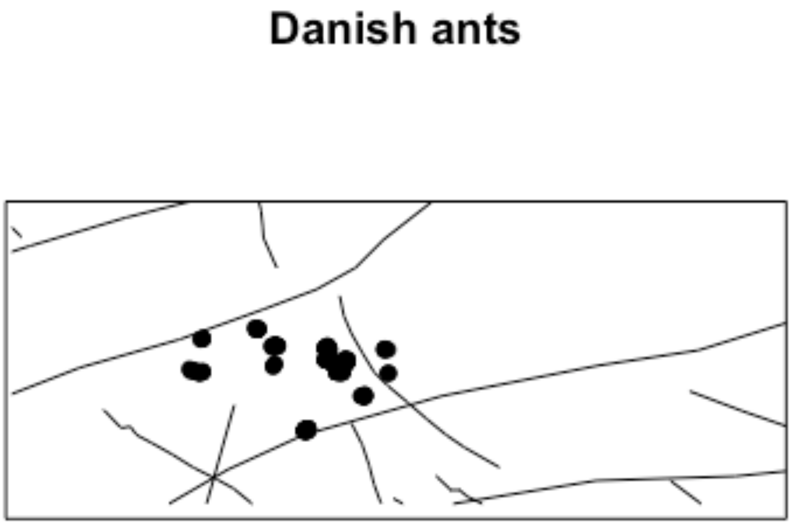


```
#let's get turn faults into a covariate that is a function of spatial
location. A reasonable first step is the distance from any point to a fault.

cov.faults <- distmap(dk.psp.faults.2)
plot(dk.psp.faults.2, lwd=2, man="")
contour(cov.faults, add=TRUE)
```

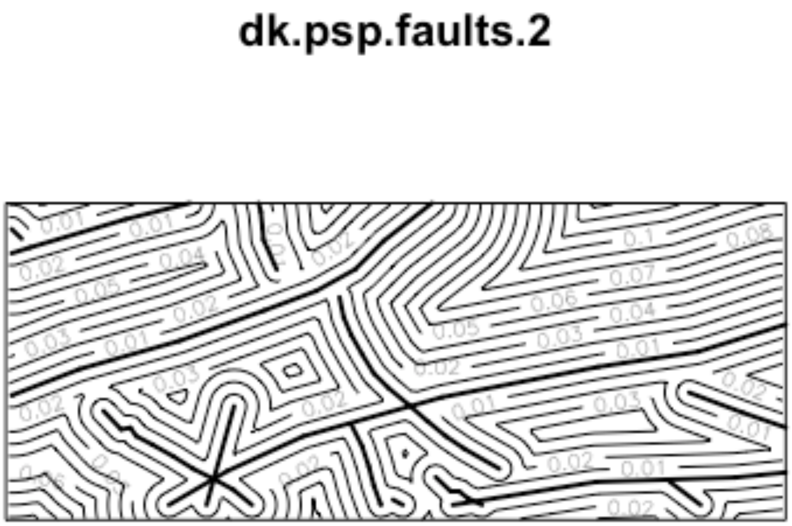


```
#relative distribution of ants to faults
#We examine how intensity of the point process (ants, no ants) varies as a
function rho of the covariate
#Because the pattern is inhomogeneous, we use a kernel smoother
tiff("Fig2AB.tiff", height = 4, width = 8, units="in", res=600)
par(mfrow=c(1,2))
plot(rhohat(dk.ppp.ants.2a, cov.faults), xlab="Distance to nearest fault",
ylim=c(0,5000), xlim=c(0,.12), main="ant mounds", col= "#e41a1c", lwd=2,
legend=F)

plot(rhohat(dk.ppp.ants.2na, cov.faults), xlab="Distance to nearest fault",
ylim=c(0,5000), xlim=c(0,.12), main="no ants", col="#377eb8", lwd=2, legend=F
)

dev.off()

## quartz_off_screen
##                 2

#test it....
rho.ants <- rhohat(dk.ppp.ants.2a, cov.faults)
rho.no.ants <- rhohat(dk.ppp.ants.2na, cov.faults)
ks.test(rho.ants$rho, rho.no.ants$rho)
```

```
## Warning in ks.test(rho.ants$rho, rho.no.ants$rho): p-value will be
## approximate in the presence of ties

##
##  Two-sample Kolmogorov-Smirnov test
##
## data:  rho.ants$rho and rho.no.ants$rho
## D = 0.36914, p-value < 2.2e-16
## alternative hypothesis: two-sided
```

make a supplementary plot to show differences between fault directionality and ant associations

```
#no ants to compare against
par(mfrow=c(2,3))
plot(rhohat(dk.ppp.ants.2na, cov.faults), xlab="Distance to nearest fault",
ylim=c(0,9500), xlim=c(0,.12), main="no ants", col="#377eb8", lwd=2, legend=F
)
#### Now lets consider the directionality of the faults
#Lets consider major faults
dk.faults <- read.csv("Faults_10_26.csv", header=T) #this is the file for
geological faults

#subset this dataset to include only normal faults
dk.faults<-dk.faults[which(dk.faults$Fault_Tren =="NW-SE"),]
summary(dk.faults)
```

```
##        Id       x_start          y_start          x_end
##  Min.   :0   Min.   : 7.784   Min.   :55.43   Min.   : 7.925
##  1st Qu.:0   1st Qu.: 8.505   1st Qu.:56.37   1st Qu.: 8.523
##  Median :0   Median : 8.795   Median :56.79   Median : 8.844
##  Mean   :0   Mean   : 9.007   Mean   :56.76   Mean   : 9.036
##  3rd Qu.:0   3rd Qu.: 9.454   3rd Qu.:57.06   3rd Qu.: 9.454
##  Max.   :0   Max.   :10.922   Max.   :57.90   Max.   :10.950
##
##      y_end                Fault_Type           Fault_Age     Fault_Tren
##  Min.   :55.54   Fault        : 64   Pre-Quaternary: 33   NW-SE  :228
##  1st Qu.:56.35   Major Fault :122   NA's          :195   ENE-WSW:  0
##  Median :56.79   Normal Fault: 42                        N-S    :  0
##  Mean   :56.74                                           NE-SW  :  0
##  3rd Qu.:57.04                                           NNE-SSW:  0
##  Max.   :57.89                                           NNW-SSE:  0
##                                                          (Other):  0
##    Direction                                   Author
##  Min.   :-326.2   2015 GEUS Server                  :74
##  1st Qu.:-326.2   2010 Pedersen Gravesen            :33
##  Median :-326.2   2010 Pedersen-Gravensen           :20
##  Mean   :-326.2   1998 Veijbaek/2008 Petersen et al.: 1
##  3rd Qu.:-326.2   1999 Veijbaek/2008 Petersen et al.: 1
##  Max.   :-326.2   2003 Veijbaek/2008 Petersen et al.: 1
##                   (Other)                           :98
```

```
#Start by identifying the extent ("window") of the ant data
dk.owin.ants <- owin(c(min(dk.ants$x)-.05, max(dk.ants$x)+.05),
c(min(dk.ants$y)-.05, max(dk.ants$y)+.05) )

#create a "point pattern object" out of dk.ants
dk.ppp.ants <- ppp(dk.ants$x, dk.ants$y, window=dk.owin.ants,
marks=dk.ants$SP_ID)

#now creat a "point segment object" out of dk.faults
dk.owin.faults <- owin(c(min(dk.faults$x_start, dk.faults$x_end),
max(dk.faults$x_start, dk.faults$x_end)), c(min(dk.faults$y_start,
dk.faults$y_end),    max(dk.faults$y_start, dk.faults$y_end)))

dk.psp.faults <- psp(dk.faults$x_start, dk.faults$y_start, dk.faults$x_end,
dk.faults$y_end, window=dk.owin.faults)

dk.psp.faults.1 <- dk.psp.faults[dk.owin.ants]

str(dk.ppp.ants)

## List of 6
##  $ window    :List of 4
##   ..$ type  : chr "rectangle"
##   ..$ xrange: num [1:2] 8.16 9.08
##   ..$ yrange: num [1:2] 56.4 57.2
##   ..$ units :List of 3
##   .. ..$ singular  : chr "unit"
##   .. ..$ plural    : chr "units"
##   .. ..$ multiplier: num 1
##   .. ..- attr(*, "class")= chr "units"
##   ..- attr(*, "class")= chr "owin"
##  $ n         : int 453
##  $ x         : num [1:453] 8.21 8.21 8.22 8.23 8.23 ...
##  $ y         : num [1:453] 56.5 56.4 56.5 56.6 56.5 ...
##  $ markformat: chr "vector"
##  $ marks     : Factor w/ 6 levels "","fusca","none",..: 3 3 3 3 3 3 3 3 3
3 ...
##  - attr(*, "class")= chr "ppp"

str(dk.psp.faults.1)

## List of 4
##  $ ends      :'data.frame':  66 obs. of  4 variables:
##   ..$ x0: num [1:66] 8.54 9.03 9 8.97 8.93 ...
##   ..$ y0: num [1:66] 56.4 57 57 57 57.1 ...
##   ..$ x1: num [1:66] 8.56 9.06 9.03 9 8.97 ...
##   ..$ y1: num [1:66] 56.4 57 57 57 57 ...
##  $ window    :List of 4
##   ..$ type  : chr "rectangle"
##   ..$ xrange: num [1:2] 8.16 9.08
```

```
##   ..$ yrange: num [1:2] 56.4 57.2
##   ..$ units :List of 3
##   .. ..$ singular  : chr "unit"
##   .. ..$ plural    : chr "units"
##   .. ..$ multiplier: num 1
##   .. ..- attr(*, "class")= chr "units"
##   ..- attr(*, "class")= chr "owin"
##  $ n         : int 66
##  $ markformat: chr "none"
##  - attr(*, "class")= chr [1:2] "psp" "list"
```

```
#clip it
str(dk.owin.ants)
```

```
## List of 4
##  $ type  : chr "rectangle"
##  $ xrange: num [1:2] 8.16 9.08
##  $ yrange: num [1:2] 56.4 57.2
##  $ units :List of 3
##   ..$ singular  : chr "unit"
##   ..$ plural    : chr "units"
##   ..$ multiplier: num 1
##   ..- attr(*, "class")= chr "units"
##  - attr(*, "class")= chr "owin"
```

```
dk.owin.ants.2 <- dk.owin.ants

#Thy clump
dk.owin.ants$yrange <- c(56.8, 57.2)
dk.owin.ants$xrange <-c(8.4, 9.08)

#Klosterhede clump
dk.owin.ants.2$yrange <- c(56.34, 56.6)
dk.owin.ants.2$xrange <-c(8.16, 8.8)

dk.ppp.ants.2 <- dk.ppp.ants[dk.owin.ants.2]
dk.psp.faults.2 <- dk.psp.faults.1[dk.owin.ants.2]

dk.ppp.ants.2a <-unmark(dk.ppp.ants.2[dk.ppp.ants.2$marks != "none"])

dk.ppp.ants.2na <-unmark(dk.ppp.ants.2[dk.ppp.ants.2$marks == "none"])

#let's get turn faults into a covariate that is a function of spatial
location. A reasonable first step is the distance from any point to a fault.

cov.faults <- distmap(dk.psp.faults.2)

#relative distribution of ants to faults
#We examine how intensity of the point process (ants, no ants) varies as a
function rho of the covariate
```

```
#Because the pattern is inhomogeneous, we use a kernel smoother

plot(rhohat(dk.ppp.ants.2a, cov.faults), xlab="Distance to nearest fault",
ylim=c(0,11500), xlim=c(0,.12), main="NW-SE", col= "#e41a1c", lwd=2,
legend=F)

#test it....
rho.ants <- rhohat(dk.ppp.ants.2a, cov.faults)
rho.no.ants <- rhohat(dk.ppp.ants.2na, cov.faults)
ks.test(rho.ants$rho, rho.no.ants$rho)
```

```
## Warning in ks.test(rho.ants$rho, rho.no.ants$rho): p-value will be
## approximate in the presence of ties
```

```
##
##  Two-sample Kolmogorov-Smirnov test
##
## data:  rho.ants$rho and rho.no.ants$rho
## D = 0.42188, p-value < 2.2e-16
## alternative hypothesis: two-sided
```

```
#test it....
rho.ants <- rhohat(dk.ppp.ants.2a, cov.faults)
rho.no.ants <- rhohat(dk.ppp.ants.2na, cov.faults)
ks.test(rho.ants$rho, rho.no.ants$rho)
```

```
## Warning in ks.test(rho.ants$rho, rho.no.ants$rho): p-value will be
## approximate in the presence of ties
```

```
##
##  Two-sample Kolmogorov-Smirnov test
##
## data:  rho.ants$rho and rho.no.ants$rho
## D = 0.42188, p-value < 2.2e-16
## alternative hypothesis: two-sided
```

```
#strong association between normal faults and ant occurances. Ranging from 4
to 10 m from a normal fault

#strong association between normal faults and ant occurances. Ranging from 4
to 10 m from a normal fault
dk.faults <- read.csv("Faults_10_26.csv", header=T) #this is the file for
geological faults
summary (dk.faults)
```

```
##        Id       x_start          y_start          x_end
##  Min.   :0   Min.   : 7.711   Min.   :55.43   Min.   : 7.635
##  1st Qu.:0   1st Qu.: 8.552   1st Qu.:56.41   1st Qu.: 8.583
##  Median :0   Median : 8.969   Median :56.77   Median : 9.013
##  Mean   :0   Mean   : 9.071   Mean   :56.74   Mean   : 9.106
##  3rd Qu.:0   3rd Qu.: 9.483   3rd Qu.:57.07   3rd Qu.: 9.529
```

```
##  Max.   :0   Max.   :10.973   Max.   :57.90   Max.   :10.950  
##                                                                
##      y_end                 Fault_Type            Fault_Age     Fault_Tren 
##  Min.   :55.44   Fault        :215   Pre-Quaternary: 38   NW-SE   :228  
##  1st Qu.:56.40   Major Fault  :238   NA's          :564   WNW-ESE:140  
##  Median :56.74   Normal Fault:149                         NNW-SSE: 93  
##  Mean   :56.73                                            NE-SW  : 88  
##  3rd Qu.:57.05                                            NNE-SSW: 40  
##  Max.   :57.90                                            ENE-WSW:  7  
##                                                           (Other):  6  
##    Direction                                        Author   
##  Min.   :-348.8   2015 GEUS Server                     :179  
##  1st Qu.:-326.2   2010 Pedersen-Gravensen              : 54  
##  Median :-326.2   2010 Pedersen Gravesen               : 38  
##  Mean   :-261.6   1997 Veijbaek/2008 Petersen et al.:  1  
##  3rd Qu.:-303.8   1998 Veijbaek/2008 Petersen et al.:  1  
##  Max.   :   0.0   1999 Veijbaek/2008 Petersen et al.:  1  
##                   (Other)                              :328  
```

```
#subset this dataset to include only normal faults
dk.faults<-dk.faults[which(dk.faults$Fault_Tren =="NNW-SSE"),]
summary(dk.faults)
```

```
##        Id       x_start          y_start          x_end       
##  Min.   :0   Min.   : 7.820   Min.   :55.67   Min.   : 7.833  
##  1st Qu.:0   1st Qu.: 8.474   1st Qu.:56.52   1st Qu.: 8.490  
##  Median :0   Median : 9.115   Median :56.83   Median : 9.129  
##  Mean   :0   Mean   : 9.075   Mean   :56.74   Mean   : 9.081  
##  3rd Qu.:0   3rd Qu.: 9.369   3rd Qu.:57.00   3rd Qu.: 9.371  
##  Max.   :0   Max.   :10.307   Max.   :57.47   Max.   :10.290  
##                                                                
##      y_end                 Fault_Type           Fault_Age    Fault_Tren 
##  Min.   :55.71   Fault        :21   Pre-Quaternary: 0   NNW-SSE:93  
##  1st Qu.:56.51   Major Fault  :49   NA's          :93   ENE-WSW: 0  
##  Median :56.79   Normal Fault:23                        N-S    : 0  
##  Mean   :56.72                                          NE-SW  : 0  
##  3rd Qu.:56.97                                          NNE-SSW: 0  
##  Max.   :57.41                                          NW_SE  : 0  
##                                                         (Other): 0  
##    Direction                                        Author  
##  Min.   :-348.8   2015 GEUS Server                     :49  
##  1st Qu.:-348.8   2010 Pedersen-Gravensen              : 7  
##  Median :-348.8   1997 Veijbaek/2008 Petersen et al.: 1  
##  Mean   :-348.8   2017 Veijbaek/2008 Petersen et al.: 1  
##  3rd Qu.:-348.8   2019 Veijbaek/2008 Petersen et al.: 1  
##  Max.   :-348.8   2025 Veijbaek/2008 Petersen et al.: 1  
##                   (Other)                              :33  
```

```
#Start by identifying the extent ("window") of the ant data
dk.owin.ants <- owin(c(min(dk.ants$x)-.05, max(dk.ants$x)+.05),
```

```
c(min(dk.ants$y)-.05, max(dk.ants$y)+.05) )

#create a "point pattern object" out of dk.ants
dk.ppp.ants <- ppp(dk.ants$x, dk.ants$y, window=dk.owin.ants,
marks=dk.ants$SP_ID)

#now creat a "point segment object" out of dk.faults
dk.owin.faults <- owin(c(min(dk.faults$x_start, dk.faults$x_end),
max(dk.faults$x_start, dk.faults$x_end)), c(min(dk.faults$y_start,
dk.faults$y_end),    max(dk.faults$y_start, dk.faults$y_end)))

dk.psp.faults <- psp(dk.faults$x_start, dk.faults$y_start, dk.faults$x_end,
dk.faults$y_end, window=dk.owin.faults)

dk.psp.faults.1 <- dk.psp.faults[dk.owin.ants]

str(dk.ppp.ants)

## List of 6
##  $ window    :List of 4
##   ..$ type  : chr "rectangle"
##   ..$ xrange: num [1:2] 8.16 9.08
##   ..$ yrange: num [1:2] 56.4 57.2
##   ..$ units :List of 3
##   .. ..$ singular  : chr "unit"
##   .. ..$ plural    : chr "units"
##   .. ..$ multiplier: num 1
##   .. ..- attr(*, "class")= chr "units"
##   ..- attr(*, "class")= chr "owin"
##  $ n         : int 453
##  $ x         : num [1:453] 8.21 8.21 8.22 8.23 8.23 ...
##  $ y         : num [1:453] 56.5 56.4 56.5 56.6 56.5 ...
##  $ markformat: chr "vector"
##  $ marks     : Factor w/ 6 levels "","fusca","none",..: 3 3 3 3 3 3 3 3 3
3 ...
##  - attr(*, "class")= chr "ppp"

str(dk.psp.faults.1)

## List of 4
##  $ ends      :'data.frame':  34 obs. of  4 variables:
##   ..$ x0: num [1:34] 8.37 9.02 9.02 9.02 9.03 ...
##   ..$ y0: num [1:34] 56.6 57.2 57.2 57.1 57.1 ...
##   ..$ x1: num [1:34] 8.38 9.02 9.02 9.03 9.03 ...
##   ..$ y1: num [1:34] 56.5 57.2 57.1 57.1 57.1 ...
##  $ window    :List of 4
##   ..$ type  : chr "rectangle"
##   ..$ xrange: num [1:2] 8.16 9.08
##   ..$ yrange: num [1:2] 56.4 57.2
##   ..$ units :List of 3
```

```
##   .. ..$ singular  : chr "unit"
##   .. ..$ plural    : chr "units"
##   .. ..$ multiplier: num 1
##   .. ..- attr(*, "class")= chr "units"
##   ..- attr(*, "class")= chr "owin"
##  $ n         : int 34
##  $ markformat: chr "none"
##  - attr(*, "class")= chr [1:2] "psp" "list"
```

```
#clip it
str(dk.owin.ants)
```

```
## List of 4
##  $ type  : chr "rectangle"
##  $ xrange: num [1:2] 8.16 9.08
##  $ yrange: num [1:2] 56.4 57.2
##  $ units :List of 3
##   ..$ singular  : chr "unit"
##   ..$ plural    : chr "units"
##   ..$ multiplier: num 1
##   ..- attr(*, "class")= chr "units"
##  - attr(*, "class")= chr "owin"
```

```
dk.owin.ants.2 <- dk.owin.ants

#Thy clump
dk.owin.ants$yrange <- c(56.8, 57.2)
dk.owin.ants$xrange <-c(8.4, 9.08)

#Klosterhede clump
dk.owin.ants.2$yrange <- c(56.34, 56.6)
dk.owin.ants.2$xrange <-c(8.16, 8.8)

dk.ppp.ants.2 <- dk.ppp.ants[dk.owin.ants.2]
dk.psp.faults.2 <- dk.psp.faults.1[dk.owin.ants.2]

dk.ppp.ants.2a <-unmark(dk.ppp.ants.2[dk.ppp.ants.2$marks != "none"])

dk.ppp.ants.2na <-unmark(dk.ppp.ants.2[dk.ppp.ants.2$marks == "none"])

#let's get turn faults into a covariate that is a function of spatial
location. A reasonable first step is the distance from any point to a fault.

cov.faults <- distmap(dk.psp.faults.2)

#relative distribution of ants to faults
#We examine how intensity of the point process (ants, no ants) varies as a
function rho of the covariate
#Because the pattern is inhomogeneous, we use a kernel smoother
plot(rhohat(dk.ppp.ants.2a, cov.faults), xlab="Distance to nearest fault",
```

```
ylim=c(0,11500), xlim=c(0,.12), main="NNW-SSE", col= "#e41a1c", lwd=2,
legend=F)

#test it....
rho.ants <- rhohat(dk.ppp.ants.2a, cov.faults)
rho.no.ants <- rhohat(dk.ppp.ants.2na, cov.faults)
ks.test(rho.ants$rho, rho.no.ants$rho)

## Warning in ks.test(rho.ants$rho, rho.no.ants$rho): p-value will be
## approximate in the presence of ties

##
##  Two-sample Kolmogorov-Smirnov test
##
## data:  rho.ants$rho and rho.no.ants$rho
## D = 0.59375, p-value < 2.2e-16
## alternative hypothesis: two-sided

#strong association between normal faults and ant occurances. Ranging from 4
to 10 m from a normal fault
dk.faults <- read.csv("Faults_10_26.csv", header=T) #this is the file for
geological faults
summary (dk.faults)

##        Id       x_start          y_start          x_end
##  Min.   :0   Min.   : 7.711   Min.   :55.43   Min.   : 7.635
##  1st Qu.:0   1st Qu.: 8.552   1st Qu.:56.41   1st Qu.: 8.583
##  Median :0   Median : 8.969   Median :56.77   Median : 9.013
##  Mean   :0   Mean   : 9.071   Mean   :56.74   Mean   : 9.106
##  3rd Qu.:0   3rd Qu.: 9.483   3rd Qu.:57.07   3rd Qu.: 9.529
##  Max.   :0   Max.   :10.973   Max.   :57.90   Max.   :10.950
##
##      y_end               Fault_Type           Fault_Age     Fault_Tren
##  Min.   :55.44   Fault        :215   Pre-Quaternary: 38   NW-SE  :228
##  1st Qu.:56.40   Major Fault :238   NA's          :564   WNW-ESE:140
##  Median :56.74   Normal Fault:149                        NNW-SSE: 93
##  Mean   :56.73                                           NE-SW  : 88
##  3rd Qu.:57.05                                           NNE-SSW: 40
##  Max.   :57.90                                           ENE-WSW:  7
##                                                          (Other):  6
##    Direction                                       Author
##  Min.   :-348.8   2015 GEUS Server                    :179
##  1st Qu.:-326.2   2010 Pedersen-Gravensen             : 54
##  Median :-326.2   2010 Pedersen Gravesen              : 38
##  Mean   :-261.6   1997 Veijbaek/2008 Petersen et al.:  1
##  3rd Qu.:-303.8   1998 Veijbaek/2008 Petersen et al.:  1
##  Max.   :   0.0   1999 Veijbaek/2008 Petersen et al.:  1
##                   (Other)                           :328
```

```r
#subset this dataset to include only normal faults
dk.faults<-dk.faults[which(dk.faults$Fault_Tren =="NE-SW"),]
summary(dk.faults)

##          Id        x_start          y_start           x_end      
##  Min.   :0   Min.   :7.711   Min.   :55.48   Min.   :7.635  
##  1st Qu.:0   1st Qu.:8.261   1st Qu.:56.42   1st Qu.:8.301  
##  Median :0   Median :8.690   Median :56.55   Median :8.696  
##  Mean   :0   Mean   :8.668   Mean   :56.59   Mean   :8.697  
##  3rd Qu.:0   3rd Qu.:9.138   3rd Qu.:56.93   3rd Qu.:9.138  
##  Max.   :0   Max.   :9.606   Max.   :57.13   Max.   :9.660  
##                                                             
##      y_end               Fault_Type           Fault_Age    Fault_Tren
##  Min.   :55.59   Fault        :50   Pre-Quaternary: 5   NE-SW  :88  
##  1st Qu.:56.41   Major Fault :20   NA's          :83   ENE-WSW: 0  
##  Median :56.55   Normal Fault:18                       N-S    : 0  
##  Mean   :56.61                                          NNE-SSW: 0  
##  3rd Qu.:56.95                                          NNW-SSE: 0  
##  Max.   :57.14                                          NW_SE  : 0  
##                                                         (Other): 0  
##    Direction                                    Author  
##  Min.   :-56.25   2015 GEUS Server                :14  
##  1st Qu.:-56.25   2010 Pedersen Gravesen          : 5  
##  Median :-56.25   2010 Pedersen-Gravensen         : 2  
##  Mean   :-56.25   2000 Veijbaek/2008 Petersen et al.: 1  
##  3rd Qu.:-56.25   2008 Veijbaek/2008 Petersen et al.: 1  
##  Max.   :-56.25   2009 Veijbaek/2008 Petersen et al.: 1  
##                   (Other)                         :64  

#Start by identifying the extent ("window") of the ant data
dk.owin.ants <- owin(c(min(dk.ants$x)-.05, max(dk.ants$x)+.05),
c(min(dk.ants$y)-.05, max(dk.ants$y)+.05) )

#create a "point pattern object" out of dk.ants
dk.ppp.ants <- ppp(dk.ants$x, dk.ants$y, window=dk.owin.ants,
marks=dk.ants$SP_ID)

#now creat a "point segment object" out of dk.faults
dk.owin.faults <- owin(c(min(dk.faults$x_start, dk.faults$x_end),
max(dk.faults$x_start, dk.faults$x_end)), c(min(dk.faults$y_start,
dk.faults$y_end),    max(dk.faults$y_start, dk.faults$y_end)))

dk.psp.faults <- psp(dk.faults$x_start, dk.faults$y_start, dk.faults$x_end,
dk.faults$y_end, window=dk.owin.faults)

dk.psp.faults.1 <- dk.psp.faults[dk.owin.ants]

str(dk.ppp.ants)
```

```
## List of 6
##  $ window    :List of 4
##   ..$ type  : chr "rectangle"
##   ..$ xrange: num [1:2] 8.16 9.08
##   ..$ yrange: num [1:2] 56.4 57.2
##   ..$ units :List of 3
##   .. ..$ singular  : chr "unit"
##   .. ..$ plural    : chr "units"
##   .. ..$ multiplier: num 1
##   .. ..- attr(*, "class")= chr "units"
##   ..- attr(*, "class")= chr "owin"
##  $ n         : int 453
##  $ x         : num [1:453] 8.21 8.21 8.22 8.23 8.23 ...
##  $ y         : num [1:453] 56.5 56.4 56.5 56.6 56.5 ...
##  $ markformat: chr "vector"
##  $ marks     : Factor w/ 6 levels "","fusca","none",..: 3 3 3 3 3 3 3 3 3
3 ...
##  - attr(*, "class")= chr "ppp"
```

```
str(dk.psp.faults.1)
```

```
## List of 4
##  $ ends      :'data.frame':  46 obs. of  4 variables:
##   ..$ x0: num [1:46] 8.48 8.73 8.62 8.59 8.56 ...
##   ..$ y0: num [1:46] 57.1 57.1 57.1 57.1 57.1 ...
##   ..$ x1: num [1:46] 8.53 8.76 8.68 8.62 8.59 ...
##   ..$ y1: num [1:46] 57.1 57.1 57.1 57.1 57.1 ...
##  $ window    :List of 4
##   ..$ type  : chr "rectangle"
##   ..$ xrange: num [1:2] 8.16 9.08
##   ..$ yrange: num [1:2] 56.4 57.2
##   ..$ units :List of 3
##   .. ..$ singular  : chr "unit"
##   .. ..$ plural    : chr "units"
##   .. ..$ multiplier: num 1
##   .. ..- attr(*, "class")= chr "units"
##   ..- attr(*, "class")= chr "owin"
##  $ n         : int 46
##  $ markformat: chr "none"
##  - attr(*, "class")= chr [1:2] "psp" "list"
```

```
#clip it
str(dk.owin.ants)
```

```
## List of 4
##  $ type  : chr "rectangle"
##  $ xrange: num [1:2] 8.16 9.08
##  $ yrange: num [1:2] 56.4 57.2
##  $ units :List of 3
##   ..$ singular  : chr "unit"
##   ..$ plural    : chr "units"
```

```
##   ..$ multiplier: num 1
##   ..- attr(*, "class")= chr "units"
##  - attr(*, "class")= chr "owin"
```

```
dk.owin.ants.2 <- dk.owin.ants

#Thy clump
dk.owin.ants$yrange <- c(56.8, 57.2)
dk.owin.ants$xrange <-c(8.4, 9.08)

#Klosterhede clump
dk.owin.ants.2$yrange <- c(56.34, 56.6)
dk.owin.ants.2$xrange <-c(8.16, 8.8)

dk.ppp.ants.2 <- dk.ppp.ants[dk.owin.ants.2]
dk.psp.faults.2 <- dk.psp.faults.1[dk.owin.ants.2]

dk.ppp.ants.2a <-unmark(dk.ppp.ants.2[dk.ppp.ants.2$marks != "none"])

dk.ppp.ants.2na <-unmark(dk.ppp.ants.2[dk.ppp.ants.2$marks == "none"])

#let's get turn faults into a covariate that is a function of spatial
location. A reasonable first step is the distance from any point to a fault.

cov.faults <- distmap(dk.psp.faults.2)

#relative distribution of ants to faults
#We examine how intensity of the point process (ants, no ants) varies as a
function rho of the covariate
#Because the pattern is inhomogeneous, we use a kernel smoother
plot(rhohat(dk.ppp.ants.2a, cov.faults), xlab="Distance to nearest fault",
ylim=c(0,11500), xlim=c(0,.12), main="NE-SW", col= "#e41a1c", lwd=2,
legend=F)

#test it....
rho.ants <- rhohat(dk.ppp.ants.2a, cov.faults)
rho.no.ants <- rhohat(dk.ppp.ants.2na, cov.faults)
ks.test(rho.ants$rho, rho.no.ants$rho)
```

```
## Warning in ks.test(rho.ants$rho, rho.no.ants$rho): p-value will be
## approximate in the presence of ties

##
##  Two-sample Kolmogorov-Smirnov test
##
## data:  rho.ants$rho and rho.no.ants$rho
## D = 0.4043, p-value < 2.2e-16
## alternative hypothesis: two-sided
```

```
#strong association between normal faults and ant occurances. Ranging from 4
to 10 m from a normal fault
dk.faults <- read.csv("Faults_10_26.csv", header=T) #this is the file for
geological faults
summary (dk.faults)

##        Id       x_start          y_start          x_end
##  Min.   :0   Min.   : 7.711   Min.   :55.43   Min.   : 7.635
##  1st Qu.:0   1st Qu.: 8.552   1st Qu.:56.41   1st Qu.: 8.583
##  Median :0   Median : 8.969   Median :56.77   Median : 9.013
##  Mean   :0   Mean   : 9.071   Mean   :56.74   Mean   : 9.106
##  3rd Qu.:0   3rd Qu.: 9.483   3rd Qu.:57.07   3rd Qu.: 9.529
##  Max.   :0   Max.   :10.973   Max.   :57.90   Max.   :10.950
##
##      y_end                Fault_Type            Fault_Age     Fault_Tren
##  Min.   :55.44   Fault        :215   Pre-Quaternary: 38   NW-SE   :228
##  1st Qu.:56.40   Major Fault :238   NA's          :564   WNW-ESE:140
##  Median :56.74   Normal Fault:149                          NNW-SSE: 93
##  Mean   :56.73                                             NE-SW  : 88
##  3rd Qu.:57.05                                             NNE-SSW: 40
##  Max.   :57.90                                             ENE-WSW:  7
##                                                            (Other):  6
##    Direction                                           Author
##  Min.   :-348.8   2015 GEUS Server                       :179
##  1st Qu.:-326.2   2010 Pedersen-Gravensen                : 54
##  Median :-326.2   2010 Pedersen Gravesen                 : 38
##  Mean   :-261.6   1997 Veijbaek/2008 Petersen et al.:  1
##  3rd Qu.:-303.8   1998 Veijbaek/2008 Petersen et al.:  1
##  Max.   :   0.0   1999 Veijbaek/2008 Petersen et al.:  1
##                   (Other)                                :328

#subset this dataset to include only normal faults
dk.faults<-dk.faults[which(dk.faults$Fault_Tren =="NNE-SSW"),]
summary(dk.faults)

##        Id       x_start         y_start          x_end
##  Min.   :0   Min.   :7.742   Min.   :55.54   Min.   :7.763
##  1st Qu.:0   1st Qu.:8.962   1st Qu.:56.36   1st Qu.:8.964
##  Median :0   Median :9.195   Median :56.68   Median :9.192
##  Mean   :0   Mean   :9.157   Mean   :56.52   Mean   :9.154
##  3rd Qu.:0   3rd Qu.:9.440   3rd Qu.:56.82   3rd Qu.:9.436
##  Max.   :0   Max.   :9.777   Max.   :57.07   Max.   :9.734
##
##      y_end                Fault_Type           Fault_Age    Fault_Tren
##  Min.   :55.44   Fault        :18   Pre-Quaternary: 0   NNE-SSW:40
##  1st Qu.:56.41   Major Fault : 1   NA's          :40   ENE-WSW: 0
##  Median :56.67   Normal Fault:21                        N-S    : 0
##  Mean   :56.50                                          NE-SW  : 0
##  3rd Qu.:56.78                                          NNW-SSE: 0
##  Max.   :57.06                                          NW_SE  : 0
```

```
##                                                                 (Other): 0
##    Direction                                                  Author
##  Min.   :-33.75   2010 Pedersen-Gravensen                        : 3
##  1st Qu.:-33.75   2001 Veijbaek/2008 Petersen et al.: 1
##  Median :-33.75   2007 Veijbaek/2008 Petersen et al.: 1
##  Mean   :-33.75   2015 GEUS Server                               : 1
##  3rd Qu.:-33.75   2016 Veijbaek/2008 Petersen et al.: 1
##  Max.   :-33.75   2018 Veijbaek/2008 Petersen et al.: 1
##                   (Other)                                        :32

#Start by identifying the extent ("window") of the ant data
dk.owin.ants <- owin(c(min(dk.ants$x)-.05, max(dk.ants$x)+.05),
c(min(dk.ants$y)-.05, max(dk.ants$y)+.05) )

#create a "point pattern object" out of dk.ants
dk.ppp.ants <- ppp(dk.ants$x, dk.ants$y, window=dk.owin.ants,
marks=dk.ants$SP_ID)

#now creat a "point segment object" out of dk.faults
dk.owin.faults <- owin(c(min(dk.faults$x_start, dk.faults$x_end),
max(dk.faults$x_start, dk.faults$x_end)), c(min(dk.faults$y_start,
dk.faults$y_end),    max(dk.faults$y_start, dk.faults$y_end)))

dk.psp.faults <- psp(dk.faults$x_start, dk.faults$y_start, dk.faults$x_end,
dk.faults$y_end, window=dk.owin.faults)

dk.psp.faults.1 <- dk.psp.faults[dk.owin.ants]

str(dk.ppp.ants)

## List of 6
##  $ window    :List of 4
##   ..$ type  : chr "rectangle"
##   ..$ xrange: num [1:2] 8.16 9.08
##   ..$ yrange: num [1:2] 56.4 57.2
##   ..$ units :List of 3
##   .. ..$ singular  : chr "unit"
##   .. ..$ plural    : chr "units"
##   .. ..$ multiplier: num 1
##   .. ..- attr(*, "class")= chr "units"
##   ..- attr(*, "class")= chr "owin"
##  $ n         : int 453
##  $ x         : num [1:453] 8.21 8.21 8.22 8.23 8.23 ...
##  $ y         : num [1:453] 56.5 56.4 56.5 56.6 56.5 ...
##  $ markformat: chr "vector"
##  $ marks     : Factor w/ 6 levels "","fusca","none",..: 3 3 3 3 3 3 3 3 3
3 ...
##  - attr(*, "class")= chr "ppp"

str(dk.psp.faults.1)
```

```
## List of 4
##  $ ends      :'data.frame':  10 obs. of  4 variables:
##   ..$ x0: num [1:10] 8.72 8.93 8.9 9.03 9 ...
##   ..$ y0: num [1:10] 57.1 57 56.8 56.7 56.7 ...
##   ..$ x1: num [1:10] 8.71 8.91 8.88 9.03 9.03 ...
##   ..$ y1: num [1:10] 57.1 56.9 56.8 56.7 56.7 ...
##  $ window    :List of 4
##   ..$ type  : chr "rectangle"
##   ..$ xrange: num [1:2] 8.16 9.08
##   ..$ yrange: num [1:2] 56.4 57.2
##   ..$ units :List of 3
##   .. ..$ singular  : chr "unit"
##   .. ..$ plural    : chr "units"
##   .. ..$ multiplier: num 1
##   .. ..- attr(*, "class")= chr "units"
##   ..- attr(*, "class")= chr "owin"
##  $ n         : int 10
##  $ markformat: chr "none"
##  - attr(*, "class")= chr [1:2] "psp" "list"
```

```
#clip it
str(dk.owin.ants)
```

```
## List of 4
##  $ type  : chr "rectangle"
##  $ xrange: num [1:2] 8.16 9.08
##  $ yrange: num [1:2] 56.4 57.2
##  $ units :List of 3
##   ..$ singular  : chr "unit"
##   ..$ plural    : chr "units"
##   ..$ multiplier: num 1
##   ..- attr(*, "class")= chr "units"
##  - attr(*, "class")= chr "owin"
```

```
dk.owin.ants.2 <- dk.owin.ants

#Thy clump
dk.owin.ants$yrange <- c(56.8, 57.2)
dk.owin.ants$xrange <-c(8.4, 9.08)

#Klosterhede clump
dk.owin.ants.2$yrange <- c(56.34, 56.6)
dk.owin.ants.2$xrange <-c(8.16, 8.8)

dk.ppp.ants.2 <- dk.ppp.ants[dk.owin.ants.2]
dk.psp.faults.2 <- dk.psp.faults.1[dk.owin.ants.2]

dk.ppp.ants.2a <-unmark(dk.ppp.ants.2[dk.ppp.ants.2$marks != "none"])

dk.ppp.ants.2na <-unmark(dk.ppp.ants.2[dk.ppp.ants.2$marks == "none"])
```

```
#let's get turn faults into a covariate that is a function of spatial location. A reasonable first step is the distance from any point to a fault.

cov.faults <- distmap(dk.psp.faults.2)

#relative distribution of ants to faults
#We examine how intensity of the point process (ants, no ants) varies as a function rho of the covariate
#Because the pattern is inhomogeneous, we use a kernel smoother
plot(rhohat(dk.ppp.ants.2a, cov.faults), xlab="Distance to nearest fault", ylim=c(0,11500), xlim=c(0,.12), main="NNE-SSW", col= "#e41a1c", lwd=2, legend=F)

#test it....
rho.ants <- rhohat(dk.ppp.ants.2a, cov.faults)
rho.no.ants <- rhohat(dk.ppp.ants.2na, cov.faults)
ks.test(rho.ants$rho, rho.no.ants$rho)

## Warning in ks.test(rho.ants$rho, rho.no.ants$rho): p-value will be
## approximate in the presence of ties

##
##  Two-sample Kolmogorov-Smirnov test
##
## data:  rho.ants$rho and rho.no.ants$rho
## D = 0.73242, p-value < 2.2e-16
## alternative hypothesis: two-sided

#strong association between normal faults and ant occurances. Ranging from 4 to 10 m from a normal fault
dk.faults <- read.csv("Faults_10_26.csv", header=T) #this is the file for geological faults
summary (dk.faults)

##        Id       x_start          y_start          x_end
##  Min.   :0   Min.   : 7.711   Min.   :55.43   Min.   : 7.635
##  1st Qu.:0   1st Qu.: 8.552   1st Qu.:56.41   1st Qu.: 8.583
##  Median :0   Median : 8.969   Median :56.77   Median : 9.013
##  Mean   :0   Mean   : 9.071   Mean   :56.74   Mean   : 9.106
##  3rd Qu.:0   3rd Qu.: 9.483   3rd Qu.:57.07   3rd Qu.: 9.529
##  Max.   :0   Max.   :10.973   Max.   :57.90   Max.   :10.950
##
##      y_end              Fault_Type            Fault_Age      Fault_Tren
##  Min.   :55.44   Fault        :215   Pre-Quaternary: 38   NW-SE   :228
##  1st Qu.:56.40   Major Fault  :238   NA's          :564   WNW-ESE:140
##  Median :56.74   Normal Fault:149                        NNW-SSE: 93
##  Mean   :56.73                                           NE-SW   : 88
##  3rd Qu.:57.05                                           NNE-SSW: 40
##  Max.   :57.90                                           ENE-WSW:  7
```

```
##                                                            (Other):  6
##    Direction                                       Author
##  Min.   :-348.8   2015 GEUS Server                    :179
##  1st Qu.:-326.2   2010 Pedersen-Gravensen             : 54
##  Median :-326.2   2010 Pedersen Gravesen              : 38
##  Mean   :-261.6   1997 Veijbaek/2008 Petersen et al.:  1
##  3rd Qu.:-303.8   1998 Veijbaek/2008 Petersen et al.:  1
##  Max.   :   0.0   1999 Veijbaek/2008 Petersen et al.:  1
##                   (Other)                             :328
```

```
#subset this dataset to include only normal faults
dk.faults<-dk.faults[which(dk.faults$Fault_Tren =="WNW-ESE"),]
summary(dk.faults)
```

```
##        Id       x_start          y_start          x_end
##  Min.   :0   Min.   : 7.742   Min.   :55.54   Min.   : 7.941
##  1st Qu.:0   1st Qu.: 8.843   1st Qu.:56.44   1st Qu.: 8.875
##  Median :0   Median : 9.321   Median :57.04   Median : 9.420
##  Mean   :0   Mean   : 9.433   Mean   :56.89   Mean   : 9.508
##  3rd Qu.:0   3rd Qu.:10.074   3rd Qu.:57.24   3rd Qu.:10.153
##  Max.   :0   Max.   :10.973   Max.   :57.89   Max.   :10.940
##
##      y_end              Fault_Type          Fault_Age      Fault_Tren
##  Min.   :55.53   Fault        :55   Pre-Quaternary:  0   WNW-ESE:140
##  1st Qu.:56.43   Major Fault :46   NA's          :140   ENE-WSW:  0
##  Median :57.01   Normal Fault:39                         N-S    :  0
##  Mean   :56.87                                           NE-SW  :  0
##  3rd Qu.:57.22                                           NNE-SSW:  0
##  Max.   :57.90                                           NNW-SSE:  0
##                                                          (Other):  0
##    Direction                                       Author
##  Min.   :-303.8   2015 GEUS Server                    :41
##  1st Qu.:-303.8   2010 Pedersen-Gravensen             :22
##  Median :-303.8   2004 Veijbaek/2008 Petersen et al.: 1
##  Mean   :-303.8   2005 Veijbaek/2008 Petersen et al.: 1
##  3rd Qu.:-303.8   2015 Veijbaek/2008 Petersen et al.: 1
##  Max.   :-303.8   2021 Veijbaek/2008 Petersen et al.: 1
##                   (Other)                             :73
```

```
#Start by identifying the extent ("window") of the ant data
dk.owin.ants <- owin(c(min(dk.ants$x)-.05, max(dk.ants$x)+.05),
c(min(dk.ants$y)-.05, max(dk.ants$y)+.05) )

#create a "point pattern object" out of dk.ants
dk.ppp.ants <- ppp(dk.ants$x, dk.ants$y, window=dk.owin.ants,
marks=dk.ants$SP_ID)

#now creat a "point segment object" out of dk.faults
dk.owin.faults <- owin(c(min(dk.faults$x_start, dk.faults$x_end),
max(dk.faults$x_start, dk.faults$x_end)), c(min(dk.faults$y_start,
```

```
dk.faults$y_end),    max(dk.faults$y_start, dk.faults$y_end)))

dk.psp.faults <- psp(dk.faults$x_start, dk.faults$y_start, dk.faults$x_end,
dk.faults$y_end, window=dk.owin.faults)

dk.psp.faults.1 <- dk.psp.faults[dk.owin.ants]

str(dk.ppp.ants)

## List of 6
##  $ window    :List of 4
##   ..$ type  : chr "rectangle"
##   ..$ xrange: num [1:2] 8.16 9.08
##   ..$ yrange: num [1:2] 56.4 57.2
##   ..$ units :List of 3
##   .. ..$ singular  : chr "unit"
##   .. ..$ plural    : chr "units"
##   .. ..$ multiplier: num 1
##   .. ..- attr(*, "class")= chr "units"
##   ..- attr(*, "class")= chr "owin"
##  $ n         : int 453
##  $ x         : num [1:453] 8.21 8.21 8.22 8.23 8.23 ...
##  $ y         : num [1:453] 56.5 56.4 56.5 56.6 56.5 ...
##  $ markformat: chr "vector"
##  $ marks     : Factor w/ 6 levels "","fusca","none",..: 3 3 3 3 3 3 3 3 3
3 ...
##  - attr(*, "class")= chr "ppp"

str(dk.psp.faults.1)

## List of 4
##  $ ends      :'data.frame':  22 obs. of  4 variables:
##   ..$ x0: num [1:22] 8.81 8.92 8.96 8.99 9.01 ...
##   ..$ y0: num [1:22] 56.4 57 57.2 57.2 57.2 ...
##   ..$ x1: num [1:22] 8.85 8.98 8.99 9.01 9.02 ...
##   ..$ y1: num [1:22] 56.4 56.9 57.2 57.2 57.2 ...
##  $ window    :List of 4
##   ..$ type  : chr "rectangle"
##   ..$ xrange: num [1:2] 8.16 9.08
##   ..$ yrange: num [1:2] 56.4 57.2
##   ..$ units :List of 3
##   .. ..$ singular  : chr "unit"
##   .. ..$ plural    : chr "units"
##   .. ..$ multiplier: num 1
##   .. ..- attr(*, "class")= chr "units"
##   ..- attr(*, "class")= chr "owin"
##  $ n         : int 22
##  $ markformat: chr "none"
##  - attr(*, "class")= chr [1:2] "psp" "list"
```

```r
#clip it
str(dk.owin.ants)
```

```
## List of 4
##  $ type  : chr "rectangle"
##  $ xrange: num [1:2] 8.16 9.08
##  $ yrange: num [1:2] 56.4 57.2
##  $ units :List of 3
##   ..$ singular  : chr "unit"
##   ..$ plural    : chr "units"
##   ..$ multiplier: num 1
##   ..- attr(*, "class")= chr "units"
##  - attr(*, "class")= chr "owin"
```

```r
dk.owin.ants.2 <- dk.owin.ants

#Thy clump
dk.owin.ants$yrange <- c(56.8, 57.2)
dk.owin.ants$xrange <-c(8.4, 9.08)

#Klosterhede clump
dk.owin.ants.2$yrange <- c(56.34, 56.6)
dk.owin.ants.2$xrange <-c(8.16, 8.8)

dk.ppp.ants.2 <- dk.ppp.ants[dk.owin.ants.2]
dk.psp.faults.2 <- dk.psp.faults.1[dk.owin.ants.2]

dk.ppp.ants.2a <-unmark(dk.ppp.ants.2[dk.ppp.ants.2$marks != "none"])

dk.ppp.ants.2na <-unmark(dk.ppp.ants.2[dk.ppp.ants.2$marks == "none"])

#let's get turn faults into a covariate that is a function of spatial
location. A reasonable first step is the distance from any point to a fault.

cov.faults <- distmap(dk.psp.faults.2)

#relative distribution of ants to faults
#We examine how intensity of the point process (ants, no ants) varies as a
function rho of the covariate
#Because the pattern is inhomogeneous, we use a kernel smoother
plot(rhohat(dk.ppp.ants.2a, cov.faults), xlab="Distance to nearest fault",
ylim=c(0,11500), xlim=c(0,.12), main="WNW-ESE", col= "#e41a1c", lwd=2,
legend=F)
```

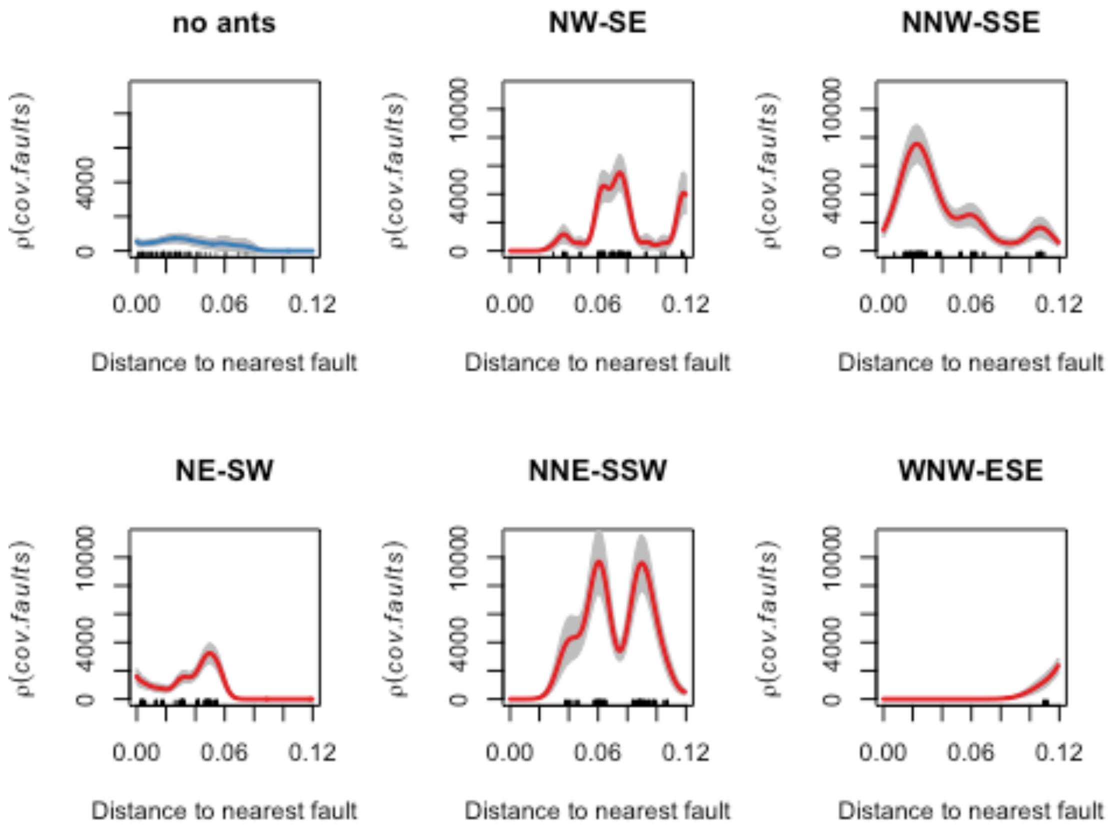


```
#test it....
rho.ants <- rhohat(dk.ppp.ants.2a, cov.faults)
rho.no.ants <- rhohat(dk.ppp.ants.2na, cov.faults)
ks.test(rho.ants$rho, rho.no.ants$rho)

## Warning in ks.test(rho.ants$rho, rho.no.ants$rho): p-value will be
## approximate in the presence of ties

##
##  Two-sample Kolmogorov-Smirnov test
##
## data:  rho.ants$rho and rho.no.ants$rho
## D = 0.58594, p-value < 2.2e-16
## alternative hypothesis: two-sided
```

In response to reviewer concerns, we now include an additional analysis of spatial clustering at the scale of the grid cell and not the ant mound. This analysis addresses the question: "Does regional nest spatial clustering associate with the presenecne of tectonic faults?""

```
#subset the dataset to show only the plots with ants (presence/absence)
sites<-read.csv ('dk_sites.csv')
sites<- sites[which (sites$PA=="1"),]
```

```r
#### Now lets consider the directionality of the faults
#Lets consider major faults
dk.faults <- read.csv("Faults_10_26.csv", header=T) #this is the file for
geological faults

#Start by identifying the extent ("window") of the ant data
dk.owin.ants <- owin(c(min(sites$x)-.05, max(sites$x)+.05), c(min(sites$y)-
.05, max(sites$y)+.05) )

#create a "point pattern object" out of dk.ants
dk.ppp.ants <- ppp(sites$x, sites$y, window=dk.owin.ants, marks=sites$PA)

#now creat a "point segment object" out of dk.faults
dk.owin.faults <- owin(c(min(dk.faults$x_start, dk.faults$x_end),
max(dk.faults$x_start, dk.faults$x_end)), c(min(dk.faults$y_start,
dk.faults$y_end),    max(dk.faults$y_start, dk.faults$y_end)))

dk.psp.faults <- psp(dk.faults$x_start, dk.faults$y_start, dk.faults$x_end,
dk.faults$y_end, window=dk.owin.faults)

dk.psp.faults.1 <- dk.psp.faults[dk.owin.ants]

str(dk.ppp.ants)

## List of 6
##  $ window    :List of 4
##   ..$ type  : chr "rectangle"
##   ..$ xrange: num [1:2] 8.26 9.08
##   ..$ yrange: num [1:2] 56.4 57.2
##   ..$ units :List of 3
##   .. ..$ singular  : chr "unit"
##   .. ..$ plural    : chr "units"
##   .. ..$ multiplier: num 1
##   .. ..- attr(*, "class")= chr "units"
##   ..- attr(*, "class")= chr "owin"
##  $ n         : int 31
##  $ x         : num [1:31] 8.88 9.03 8.96 8.97 8.99 ...
##  $ y         : num [1:31] 57 57 57.1 57.1 57.1 ...
##  $ markformat: chr "vector"
##  $ marks     : int [1:31] 1 1 1 1 1 1 1 1 1 1 ...
##  - attr(*, "class")= chr "ppp"

str(dk.psp.faults.1)

## List of 4
##  $ ends      :'data.frame':  166 obs. of  4 variables:
##   ..$ x0: num [1:166] 8.37 8.54 8.81 8.48 8.72 ...
##   ..$ y0: num [1:166] 56.6 56.4 56.4 57.1 57.1 ...
##   ..$ x1: num [1:166] 8.38 8.56 8.85 8.53 8.71 ...
##   ..$ y1: num [1:166] 56.5 56.4 56.4 57.1 57.1 ...
```

```
##  $ window    :List of 4
##   ..$ type  : chr "rectangle"
##   ..$ xrange: num [1:2] 8.26 9.08
##   ..$ yrange: num [1:2] 56.4 57.2
##   ..$ units :List of 3
##   .. ..$ singular  : chr "unit"
##   .. ..$ plural    : chr "units"
##   .. ..$ multiplier: num 1
##   .. ..- attr(*, "class")= chr "units"
##   ..- attr(*, "class")= chr "owin"
##  $ n         : int 166
##  $ markformat: chr "none"
##  - attr(*, "class")= chr [1:2] "psp" "list"
```

```
#clip it
str(dk.owin.ants)
```

```
## List of 4
##  $ type  : chr "rectangle"
##  $ xrange: num [1:2] 8.26 9.08
##  $ yrange: num [1:2] 56.4 57.2
##  $ units :List of 3
##   ..$ singular  : chr "unit"
##   ..$ plural    : chr "units"
##   ..$ multiplier: num 1
##   ..- attr(*, "class")= chr "units"
##  - attr(*, "class")= chr "owin"
```

```
dk.owin.ants.2 <- dk.owin.ants

#Thy clump
dk.owin.ants$yrange <- c(56.8, 57.2)
dk.owin.ants$xrange <-c(8.4, 9.08)

#Klosterhede clump
dk.owin.ants.2$yrange <- c(56.34, 56.6)
dk.owin.ants.2$xrange <-c(8.16, 8.8)

dk.ppp.ants.2 <- dk.ppp.ants[dk.owin.ants.2]
dk.psp.faults.2 <- dk.psp.faults.1[dk.owin.ants.2]

dk.ppp.ants.2a <-unmark(dk.ppp.ants.2[dk.ppp.ants.2$marks != "none"])

dk.ppp.ants.2na <-unmark(dk.ppp.ants.2[dk.ppp.ants.2$marks == "none"])

#let's get turn faults into a covariate that is a function of spatial
location. A reasonable first step is the distance from any point to a fault.

cov.faults <- distmap(dk.psp.faults.2)
```

```r
#relative distribution of ants to faults
#We examine how intensity of the point process (ants, no ants) varies as a
function rho of the covariate
#Because the pattern is inhomogeneous, we use a kernel smoother
plot(rhohat(dk.ppp.ants.2a, cov.faults), xlab="Distance to nearest fault",
ylim=c(0,400), xlim=c(0,.12), main="Grid- Level Analyses", col= "#e41a1c",
lwd=2, legend=T)
```

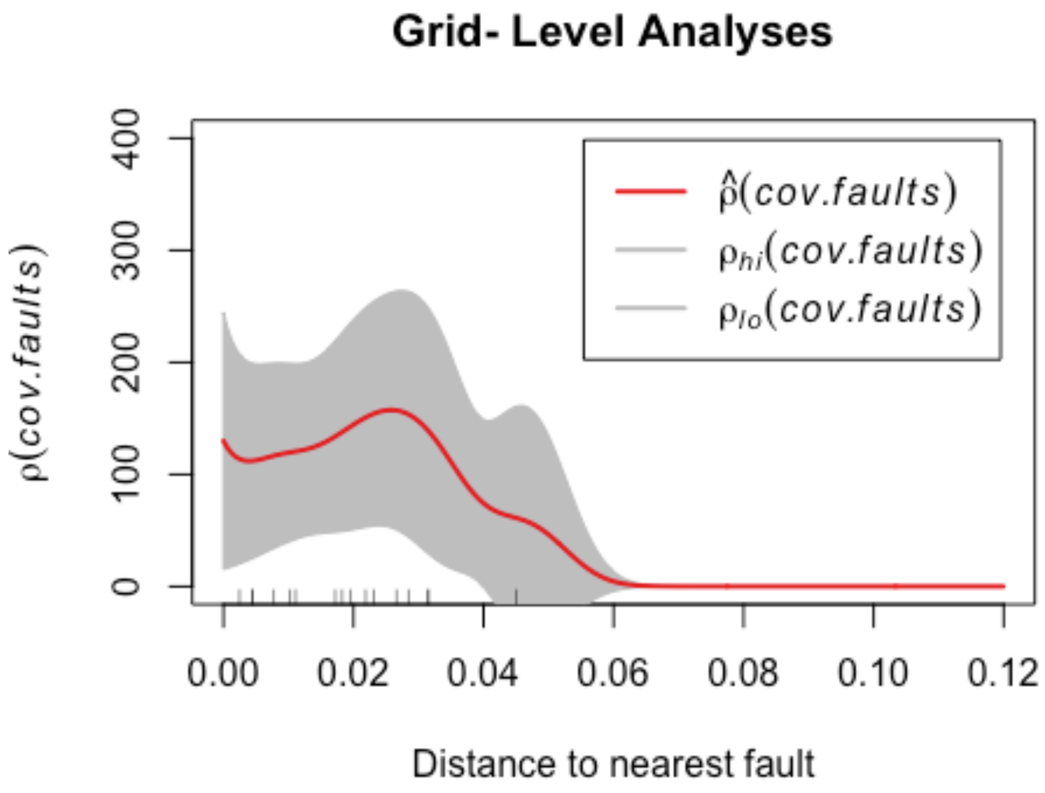


```r
#test it....
rho.ants <- rhohat(dk.ppp.ants.2a, cov.faults)
ks.test(rho.ants$rho, rho.no.ants$rho)

## Warning in ks.test(rho.ants$rho, rho.no.ants$rho): p-value will be
## approximate in the presence of ties

##
##  Two-sample Kolmogorov-Smirnov test
##
## data:  rho.ants$rho and rho.no.ants$rho
## D = 0.91016, p-value < 2.2e-16
## alternative hypothesis: two-sided

# a test of the rufa vs polyctena effect: Question: Does the spatial pattern
between F.rufa and F. polyctena differ?
```

```r
setwd("/Users/israel/Desktop/OneDrive - Lawrence University/DK Ants")
rufa<-dk.ants[which(dk.ants$SP_ID=="rufa"),]
par(mfrow=c(1,1))

#### Now lets consider the directionality of the faults
#Lets consider major faults
dk.faults <- read.csv("Faults_10_26.csv", header=T) #this is the file for geological faults

#Start by identifying the extent ("window") of the ant data
dk.owin.ants <- owin(c(min(rufa$x)-.05, max(rufa$x)+.05), c(min(rufa$y)-.05, max(rufa$y)+.05) )

#create a "point pattern object" out of dk.ants
dk.ppp.ants <- ppp(rufa$x, rufa$y, window=dk.owin.ants, marks=rufa$SP_ID)

#now creat a "point segment object" out of dk.faults
dk.owin.faults <- owin(c(min(dk.faults$x_start, dk.faults$x_end), max(dk.faults$x_start, dk.faults$x_end)), c(min(dk.faults$y_start, dk.faults$y_end),    max(dk.faults$y_start, dk.faults$y_end)))

dk.psp.faults <- psp(dk.faults$x_start, dk.faults$y_start, dk.faults$x_end, dk.faults$y_end, window=dk.owin.faults)

dk.psp.faults.1 <- dk.psp.faults[dk.owin.ants]

str(dk.ppp.ants)

## List of 6
##  $ window    :List of 4
##   ..$ type  : chr "rectangle"
##   ..$ xrange: num [1:2] 8.26 9.08
##   ..$ yrange: num [1:2] 56.4 57.1
##   ..$ units :List of 3
##   .. ..$ singular  : chr "unit"
##   .. ..$ plural    : chr "units"
##   .. ..$ multiplier: num 1
##   .. ..- attr(*, "class")= chr "units"
##   ..- attr(*, "class")= chr "owin"
##  $ n         : int 34
##  $ x         : num [1:34] 8.31 8.31 8.31 8.32 8.32 ...
##  $ y         : num [1:34] 56.5 56.5 56.5 56.5 56.5 ...
##  $ markformat: chr "vector"
##  $ marks     : Factor w/ 6 levels "","fusca","none",..: 5 5 5 5 5 5 5 5 5 5 ...
##  - attr(*, "class")= chr "ppp"

str(dk.psp.faults.1)
```

```
## List of 4
##  $ ends      :'data.frame':  159 obs. of  4 variables:
##   ..$ x0: num [1:159] 8.37 8.54 8.81 8.48 8.72 ...
##   ..$ y0: num [1:159] 56.6 56.4 56.4 57.1 57.1 ...
##   ..$ x1: num [1:159] 8.38 8.56 8.85 8.53 8.71 ...
##   ..$ y1: num [1:159] 56.5 56.4 56.4 57.1 57.1 ...
##  $ window    :List of 4
##   ..$ type  : chr "rectangle"
##   ..$ xrange: num [1:2] 8.26 9.08
##   ..$ yrange: num [1:2] 56.4 57.1
##   ..$ units :List of 3
##   .. ..$ singular  : chr "unit"
##   .. ..$ plural    : chr "units"
##   .. ..$ multiplier: num 1
##   .. ..- attr(*, "class")= chr "units"
##   ..- attr(*, "class")= chr "owin"
##  $ n         : int 159
##  $ markformat: chr "none"
##  - attr(*, "class")= chr [1:2] "psp" "list"
```

```
#clip it
str(dk.owin.ants)
```

```
## List of 4
##  $ type  : chr "rectangle"
##  $ xrange: num [1:2] 8.26 9.08
##  $ yrange: num [1:2] 56.4 57.1
##  $ units :List of 3
##   ..$ singular  : chr "unit"
##   ..$ plural    : chr "units"
##   ..$ multiplier: num 1
##   ..- attr(*, "class")= chr "units"
##  - attr(*, "class")= chr "owin"
```

```
dk.owin.ants.2 <- dk.owin.ants

#Thy clump
dk.owin.ants$yrange <- c(56.8, 57.2)
dk.owin.ants$xrange <-c(8.4, 9.08)

#Klosterhede clump
dk.owin.ants.2$yrange <- c(56.34, 56.6)
dk.owin.ants.2$xrange <-c(8.16, 8.8)

dk.ppp.ants.2 <- dk.ppp.ants[dk.owin.ants.2]
dk.psp.faults.2 <- dk.psp.faults.1[dk.owin.ants.2]

dk.ppp.ants.2b <-unmark(dk.ppp.ants.2[dk.ppp.ants.2$marks != "none"])

dk.ppp.ants.2na <-unmark(dk.ppp.ants.2[dk.ppp.ants.2$marks == "none"])
```

```r
#let's get turn faults into a covariate that is a function of spatial location. A reasonable first step is the distance from any point to a fault.

cov.faults <- distmap(dk.psp.faults.2)

#relative distribution of ants to faults
#We examine how intensity of the point process (ants, no ants) varies as a function rho of the covariate
#Because the pattern is inhomogeneous, we use a kernel smoother
plot(rhohat(dk.ppp.ants.2b, cov.faults), xlab="Distance to nearest fault", ylim=c(0,1000), xlim=c(0,.12), main="F. rufa", col= "#e41a1c", lwd=2, legend=T)
```

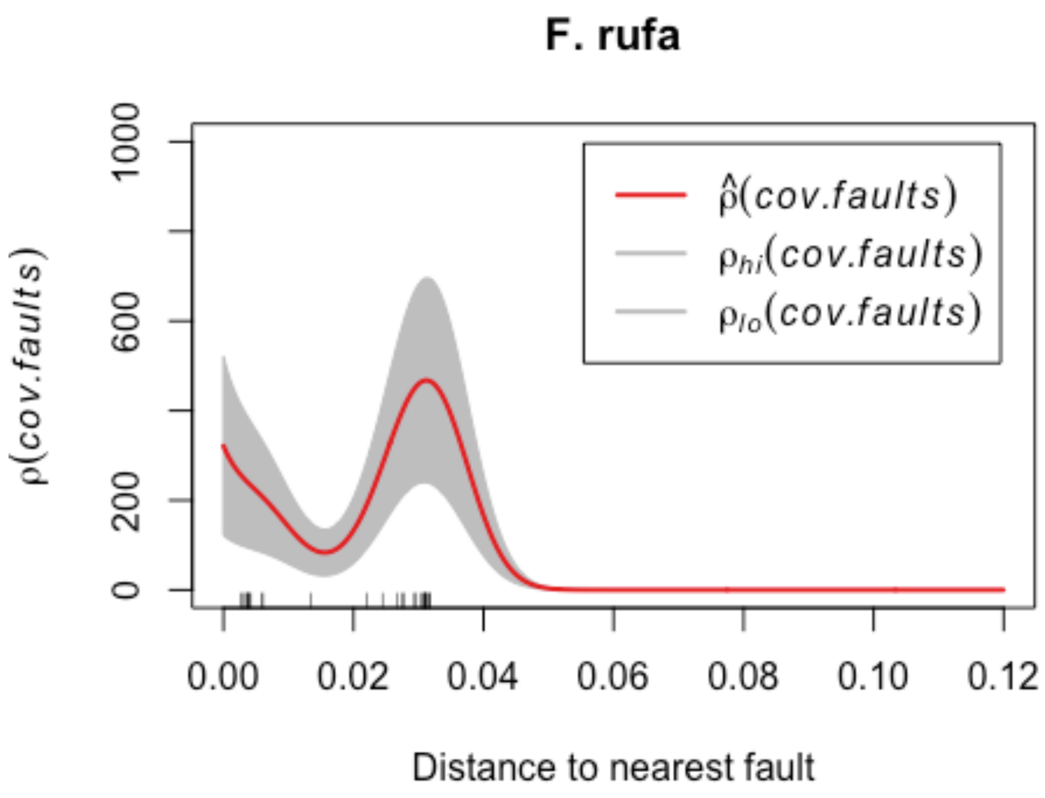


```r
#test it....
rho.ants <- rhohat(dk.ppp.ants.2a, cov.faults)
ks.test(rho.ants$rho, rho.no.ants$rho)

## Warning in ks.test(rho.ants$rho, rho.no.ants$rho): p-value will be
## approximate in the presence of ties

##
##  Two-sample Kolmogorov-Smirnov test
```

```
##
## data:  rho.ants$rho and rho.no.ants$rho
## D = 0.91016, p-value < 2.2e-16
## alternative hypothesis: two-sided
```
